# Species diversity estimation in a typical tropical forest: which phenological stage and spatial resolution are suitable?

**DOI:** 10.3389/fpls.2025.1582910

**Published:** 2025-08-18

**Authors:** Ping Zhao, Yuan Zeng, Zhaoju Zheng, Cong Xu, Jinchen Wu, Xuan Mu, Zhaofu Zhou, Junhua Chen, Tao Zhang, Dan Zhao

**Affiliations:** ^1^ Key Laboratory of Earth Observation of Hainan Province, Hainan Aerospace Information Research Institute, Wenchang, China; ^2^ Aerospace Information Research Institute, Chinese Academy of Sciences, Beijing, China; ^3^ College of Resources and Environment, University of Chinese Academy of Sciences, Beijing, China; ^4^ Hainan Jianfengling National Key Field Station for Forest Ecosystem, Research Institute of Tropical Forestry, Chinese Academy of Forestry, Guangzhou, China

**Keywords:** tropical forest, species diversity, phenology, spatial scale, sentinel-2, GF2

## Abstract

Satellite remote sensing data is essential for large-scale, timely, and repeatable monitoring of forest species diversity. While various methods have been applied to satellite-based diversity estimation at regional scales, selecting suitable sensor and monitoring period remains challenging, especially in tropical forests. This study aims to identify the optimal time window, spatial resolution, and metrics for species diversity estimation in the Jianfengling tropical forest in southern China. We constructed stepwise linear regression models for estimating Richness, Simpson, and Shannon-Wiener indices using *in-situ* species diversity and heterogeneity metrics of spectra and structure. For analyzing phenology influence, we utilized six Sentinel-2 images acquired bimonthly from January to November. For evaluating scale dependency, we resampled the GF2 image to five spatial resolutions ranging from 0.8 to 10 m. The results indicated that the suitable phenological periods for species diversity estimation were at the beginning and end of the growing season, especially September performing the best for all diversity indices. Among four types of heterogeneity metrics, spectral information consistently explained most variance in species diversity indices across all periods. The optimal spatial resolution for estimating Richness and Shannon-Wiener index was 4–5 m, which corresponded to the average tree crown size. The texture features made a significant contribution compared to other metrics. Our study highlights that species diversity monitoring is highly dependent on the spatiotemporal scales of remote sensing data. It may offer practical guidance for selecting appropriate data and methods for species diversity monitoring in tropical forests.

## 1 Introduction

Forest species diversity is an important indicator of forest ecosystem health, which plays a vital role in maintaining ecosystem services, functions, and stability (Pennekamp et al., 2018). Tropical forests, as one of the most biologically diverse ecosystems on Earth, are crucial for mitigating climate change (Csillik et al., 2019). However, they are suffering a rapid loss of biodiversity due to increasing pressures from human disturbances, climate change, and biological invasions (Montràs-Janer et al., 2024). Moreover, the dense evergreen vegetation and complex structure characteristics make it particularly challenging to estimate species diversity accurately and understand the dynamics timely (Wiegand et al., 2017). Therefore, effective methods to monitor forest species diversity across different spatial and temporal scales are urgently required for assessing biodiversity status and guiding sustainable forest management.

Traditional forest surveys could provide accurate species diversity measurements at local scale. However, limited accessibility and high costs of large-scale *in-situ* data collection lead to calls for remotely sensed monitoring as a complement (Kerr and Ostrovsky, 2003). Remote sensing approaches for species diversity estimation can be broadly divided into two categories: direct classification of species or functional types and indirect estimation based on the relationships between species diversity indices and multivariate heterogeneity indicators (Turner, 2014). Many studies have explored the relationships between spectral heterogeneity metrics and species diversity indices at landscape scale utilizing airborne hyperspectral imagery, but its limited coverage and low repeatability preclude widespread usage for monitoring species diversity across larger spatial and temporal scales (Fassnacht et al., 2022; Féret and Asner, 2014). In this context, multispectral satellite data that effectively balance spatiotemporal issues provide a unique opportunity for mapping species diversity at large spatial scales, which have been successfully applied to various ecosystems (Hernández-Stefanoni et al., 2012; Lopes et al., 2017). However, their potential in tropical forest ecosystems still requires further exploration.

Some studies have estimated tropical forest diversity using spaceborne data (Ganivet and Bloomberg, 2019). For instance, Kumar et al. (2022) calculated forest species diversity using information theory-based indices derived from Sentinel-2 imagery. Njomaba et al. (2024) explored the potential of PlanetScope to predict tropical species diversity using stepwise linear regression analysis. However, it has been observed that the relationship exhibits phenological sensitivity and scale dependency, and is strongly influenced by factors such as community composition, topography, and selected heterogeneity metrics, leading to high variability with positive, negative, or no correlation (Badourdine et al., 2022; Pangtey et al., 2023; Wang et al., 2018a). As a result, understanding and assessing the influence of phenology, scale, and various metrics on species diversity estimation is crucial for achieving more reliable monitoring in tropical forests.

Vegetation phenology refers to the periodic rhythms for growth and development of plants to adapt to interannual or seasonal changes in the environment (Richardson, 2018). An increasing number of studies have used multi-temporal images covering different phenological periods to improve tree species and diversity mapping accuracy (Blickensdörfer et al., 2024; Liu et al., 2023b; Persson et al., 2018). However, the necessity of combining multi-temporal images and the determination of optimal phenological periods for species diversity estimation is still challenged. Some studies showed improved performance with multi-temporal images, especially during the transition of the growing season (Immitzer and Atzberger, 2023; Xi et al., 2021), while others reported no advantage over single-date images (Pouteau et al., 2018; Torresani et al., 2019). Additionally, the subtle phenological dynamics of evergreen vegetation and limited cloud-free imagery in tropical regions result in gaps in understanding the impact of phenology on forest species diversity estimation. Thus, it is meaningful to identify suitable time windows for monitoring species diversity in tropical forests, further guiding the acquisition of temporally matched scenes.

Spatial scale is a central topic in both ecology and remote sensing (Gamon et al., 2020). An increasing number of studies have adopted the multi-scale, multi-source framework to explore how sensor characteristics and resolution affect species diversity estimation, using data from Sentinel-2, Landsat, RapidEye, IKONOS, and WorldView-2 (Mallinis et al., 2020; Nagendra et al., 2010; Wang et al., 2022). Some studies have shown that higher spatial resolution does not necessarily improve species diversity prediction, and medium resolution (10/15 m) may be optimal (Liu et al., 2024). High-resolution imagery can introduce intra-species spectral variance due to canopy shadows, while low-resolution images may obscure inter-species spectral variance under highly mixed conditions (Rocchini et al., 2010). Therefore, finding the optimal spatial resolution is critical. Given that satellite data with very high spatial and temporal resolution (e.g. GF2 and PlanetScope) allows for better matching of pixel size with crown size, it is essential to explore its applicability and determine the suitable spatial resolution for forest species diversity monitoring.

Variations in species resource-use and growth strategies shape environmental complexity across multiple dimensions (e.g., horizontal and vertical), which can be effectively characterized through heterogeneity metrics that integrate multi-source remote sensing information. Remote sensing-based heterogeneity metrics can be generally categorized into spectral and structural heterogeneity. The spectral heterogeneity metric is a bridge between spectral diversity and species diversity, which has been proven to affect species diversity predictions (Torresani et al., 2021). Nowadays, various metrics such as coefficient of variation (CV), convex hull area (CHA), convex hull volume (CHV), and spectral angle mapper (SAM), have been proposed to capture multidimensional spectral heterogeneity (Gholizadeh et al., 2018; Kruse et al., 1993). In addition, forest structural characteristics and their heterogeneity are considered to be proxies of forest species diversity (Ma et al., 2022; Zhao et al., 2018). Texture features, as expressions of spatial structure, also play a significant role in species diversity estimation (Liu et al., 2023a). However, no single heterogeneity metric was found to be universally applicable across all species diversity estimation scenarios. Therefore, it is essential to assess the importance of different metrics in estimating species diversity, particularly considering the variations under various phenological phases and resolutions.

In this study, we evaluated the impact of phenology and spatial resolution on species diversity estimation in a tropical rainforest using multi-temporal Sentinel-2 images and very-high-resolution GF2 images, then compared the performance of various metrics across different periods and resolutions. Specifically, we aimed to address the following questions: (i) Which is the crucial phenological stage for canopy species diversity estimation in tropical forests? (ii) What is the best spatial resolution for species diversity estimation? (iii) How do heterogeneity metrics quantitatively contribute to species diversity monitoring?

## 2 Materials and methods

### 2.1 Study area

**Figure 1:**
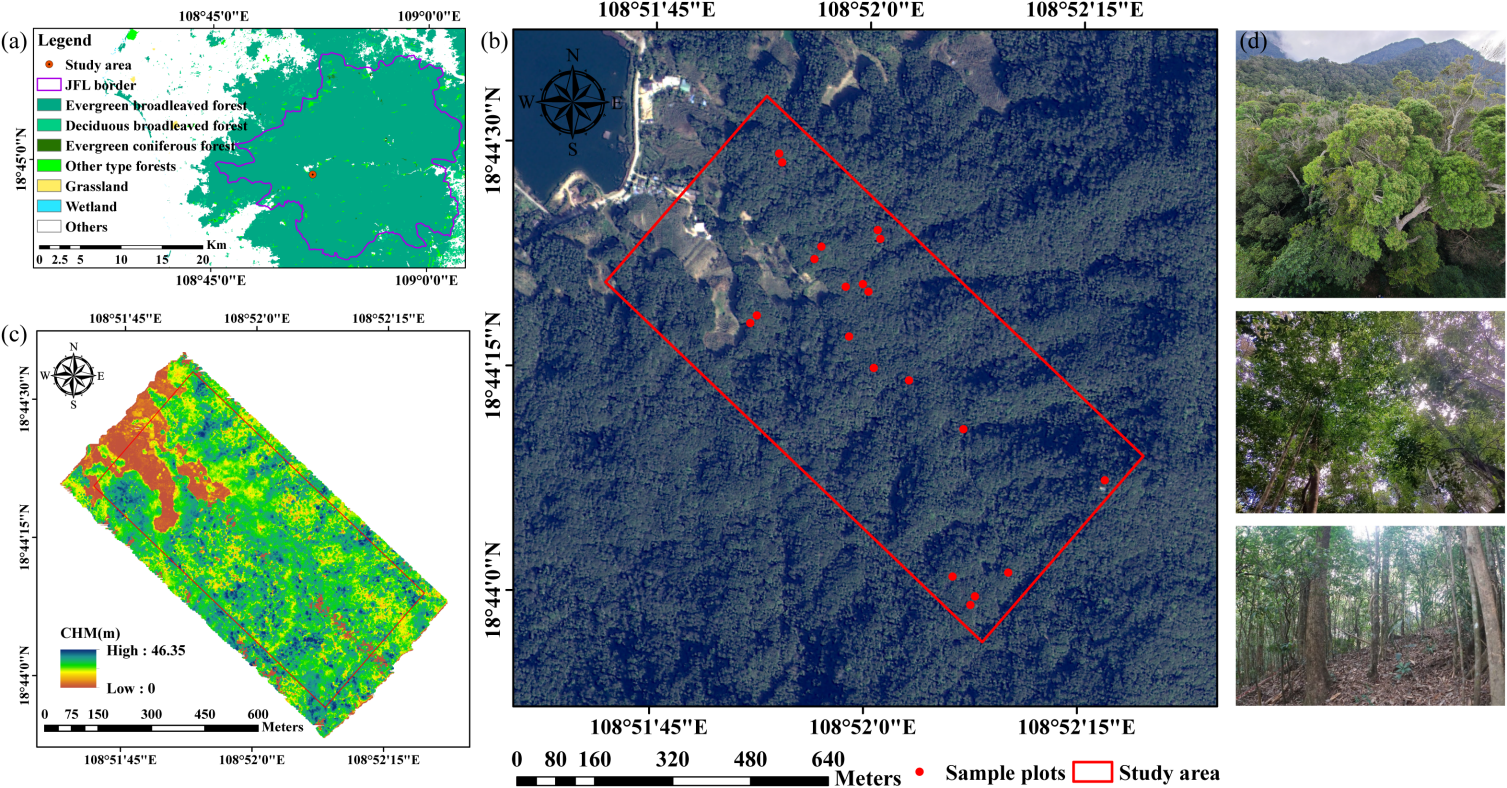
Overview of the study area and field data. **(a)** Location of the JFL National Nature Reserve with ChinaCover land cover data (Wu et al., 2017); **(b)** Location of the study area and sample plots with GF2 image; **(c)** LiDAR-derived canopy height model (CHM); **(d)** *in-situ* photos.

The study area is located in the Jianfengling National Nature Reserve in the southwest of Hainan province, China (JFL, 18°20′-18°57′N, 108°41′-109°12′E), covering an area of approximately 0.54 km^2^ (**Figure 1**). It is a typical representative of China’s tropical rainforest and belongs to the northern margin type of Asian tropical rainforest (Zhai et al., 2013). The complex and specialized structure of the rainforest provides conditions for a high diversity of plants. The dominant vegetation type is tropical montane rainforest, varying along the elevation gradient from 600 to 1200 m above sea level. This region is characterized by a tropical island monsoon climate, with an average annual temperature of 19.8°C and annual precipitation ranging from 1300 to 3700 mm. It shows distinct dry and wet seasons, with the wet season from May to October and the dry season from November to April (Xu et al., 2015; Zhu, 2017). The forest canopy across this study area comprises more than 30 dominant evergreen tree species, including *Gironniera subaequalis*, *Quercus patelliformis*, *Alniphyllum fortune*, *Castanopsis chinensis*, *Symplocos anomala*, *Dacrycarpus imbricatus*, and *Castanopsis fissa*, with varied flowering and fruiting periods (**Supplementary Figure S1**).

### 2.2 Field measurements and diversity metrics

**Figure 2:**
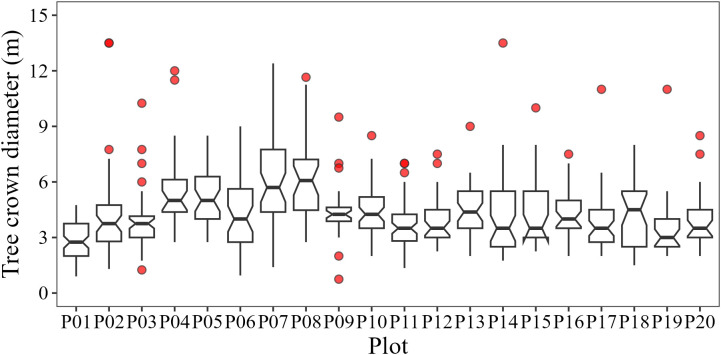
Boxplots of crown diameter of individual trees in each sample plot.

Field measurements were conducted from December 12 to 24 in 2023, across 20 sample plots (20 × 20 m) within the study area (**Figure 1**). The coordinates of the four corners of each plot were determined by integrating the Real Time Kinematic (RTK) GPS/GLONASS System with Total Station, with errors under 10 cm. In each plot, tree parameters including species name, diameters at breast height (DBH), crown classes (dominant, co-dominant, intermediate, and suppressed trees), tree height, and crown diameters in two directions (east-west and south-north) were measured to identify upper canopy trees and calculate *in-situ* species diversity. All individual trees with DBH ≥ 5 cm were recorded and upper canopies located on the first or second layers (dominant and co-dominant trees) were used to calculate species diversity. The average crown diameter was 4–5 m and some plots contained large trees (**Figure 2**).

Based on the field measurements, three commonly used species diversity metrics, namely Richness (S) (Gaston, 2000), Shannon-Wiener index (H) (Shannon, 1948), and Simpson index (D) (Simpson, 1949), were calculated within each plot to quantify different aspects of species diversity (**Supplementary Table S1**). Among them, Richness represents the number of different species. The Shannon-Wiener and Simpson indexes share certain similarities as both take species evenness into account, but the former focuses on rare species, whereas the latter emphasizes dominant species. The calculation formula of the Shannon-Wiener (H) and Simpson (D) were as Equations 1, 2:


(1) $$\begin{array}{c} H=\sum_{i=1}^{S}-p_{i}\ln p_{i} \end{array}$$

(2) $$\begin{array}{c} D=1-\sum_{i=1}^{S}p_{i}^{2} \end{array}$$

where S is the total number of species in plot, and $p_{i}$ is the proportional abundance of the species i , represented by the ratio of individuals of species i within a plot.

### 2.3 Remote sensing data

#### 2.3.1 Satellite data

Sentinel-2 can effectively capture spectral changes of vegetation across different periods due to its high revisit frequency of 5–10 days, enabling us to analyze the impact of phenology on species diversity estimation models. Considering the cloudy and rainy weather in Southern China, we downloaded all nearly cloud-free Sentinel-2 surface reflectance imagery over the study area from 2022 to 2024 to cover the entire phenological cycle. These level-2A images, consist of 13 wavebands covering visible, near- and shortwave-infrared spectra, were atmospherically corrected by ESA using the Sen2Cor algorithm (Louis et al., 2016). Finally, we obtained six Sentinel-2 images representing different phenological periods (**Table 1**). Due to cloud shading obscuring one plot in the July image, only 19 plots were used for this period. We also conducted a visual matching inspection between Sentinel-2 and UAV imagery to ensure that geolocation shift is acceptable. For analysis, we excluded the 60 m atmospheric bands since they do not contain surface information, and resampled the 20 m bands to 10 m using bilinear interpolation based on SNAP software. Then, after analyzing the NDVI distribution histogram and manually inspecting the NDVI values of non-vegetation and shadow pixels, a NDVI threshold (0.3) was applied to mask most of the non-forested pixels while preserving forest pixels from scenes during non-growing seasons.

**Table 1:** List of Sentinel-2 datasets used in this study.

Acquisition date	Sensor	Acquisition year
17 January	Sentinel-2B	2024
23 March	Sentinel-2B	2023
17 May	Sentinel-2A	2023
11 July	Sentinel-2B	2023
04 September	Sentinel-2B	2022
28 November	Sentinel-2B	2023

GF2 is an optical satellite with sub-meter spatial resolution and revisits within 5 days, which can capture vegetation information effectively. GF2 images feature one panchromatic band (0.8 m) and four VIS-NIR multispectral bands (blue, green, red, and near-infrared) with a 4 m spatial resolution, offering valuable data for our analysis of spatial scales (Zhou et al., 2020). We selected an available scene of cloud-free GF2 image (29/01/2023, download from Natural Resources Satellite Remote Sensing Cloud Service Platform) that was closest to the field sampling period and performed image pre-processing, including radiometric calibration, atmospheric correction (FLAASH module based on MODTRAN radiative transfer model), geometric correction, image fusion, and cropping, using ENVI 5.3 software (Xia et al., 2023). Finally, the NDVI threshold of 0.5 was applied to mask shadows. According to the average crown diameter, we resampled the fused image from the original (0.8 m) to coarser spatial resolutions (3 m, 4 m, 5 m, and 10 m) utilizing the nearest neighbor resampling algorithm (Liu et al., 2024). It is worth noting that there were only two scenes of GF2 data available from 2022 to 2024 due to persistent cloud and rain cover, which were insufficient to support phenological analysis. Therefore, GF2 data was only used for multi-resolution analysis.

#### 2.3.2 LiDAR data

Given the absence of spaceborne LiDAR coverage in the study area, we employed unmanned aerial vehicle (UAV) LiDAR as an alternative to analyze the importance of structural heterogeneity metrics for species diversity estimation. The UAV LiDAR data was simultaneously collected with field surveys using an FT-800H laser scanner (LuoJiaYiYun Optoelectronic Technology Co., Ltd., Wuhan, China) mounted on a DJI M300 UAV platform. The scanner provided a wide field of view of 330° and a measurement accuracy of 1 cm. The average point density was 1,058 points/m^2^. The UAV LiDAR data were denoised and filtered to generate a digital elevation model (DEM) based on classified ground points, and a digital surface model (DSM) based on the first pulse reflections. The canopy height model (CHM) was derived by subtracting the DEM from the DSM at 0.5 m resolution. UAV LiDAR data processing was performed using LiDAR360 V7.2 (GreenValley International Inc., Beijing, China). Given the subtle structural changes, a single period of structural features was sufficient for analysis.

### 2.4 Spectral and structural heterogeneity metrics

After synthesizing the rationale and applicability of commonly used metrics, four types of heterogeneity metrics were selected: spectral information metrics, spectral diversity metrics, texture features, and structural diversity metrics (**Table 2**). Spectral information metrics derived from spectral bands or vegetation indices (VIs) directly represent spectral reflectance features and highlight specific properties of vegetation. After comprehensively considering variable redundancy and spatial resolution of different bands, six representative VIs from Sentinel-2 and the corresponding three from GF2 were calculated to characterize pigment content, specific leaf area, and water content. We adopted the coefficients of these commonly used vegetation indices, as they have been validated for both sensors in previous studies (Liu et al., 2024; Xi et al., 2023; Zhou et al., 2020). For normalized difference spectral index (NDSI), we modified the band combination to reflect photosynthetic pigments and vegetation growth (Patil et al., 2007). The average values of these spectral variables (i.e., bands and VIs) were then computed for each plot. Spectral diversity metrics included CV, CHA, and SAM, where CVs were calculated based on VIs or multi-bands and CHA and SAM were calculated using all spectral bands. Eight texture features were derived from the gray level co-occurrence matrix (GLCM) based on the first principal component of Sentinel-2 and GF2 multispectral imagery. Considering the plot size, moving window sizes of GLCM were set as 27 × 27, 7 × 7, 5 × 5, 3 × 3, and 3 × 3 for the 0.8 m, 3 m, 4 m, 5 m, and 10 m resolution images respectively. For the phenological analysis based on Sentinel-2 images, we excluded the texture parameter of Cor because it was almost the same among the 20 plots. Rao’s Q index calculated based on CHM, which incorporates horizontal variation in canopy vertical structure, was selected as the representative of structural diversity (Torresani et al., 2020). We directly retrieved Rao’s Q at plot-scale using the 0.5 m CHM for temporal analysis. But in spatial scale analysis, we resampled CHM (0.8, 3, 4, 5, and 10 m) to match the optical satellite data, and then calculated Rao’s Q at plot-scale.

**Table 2:** Description of spectral and structural heterogeneity metrics.

Variables	Description	Band used	Reference
Spectral information metrics			
Bands	*Average reflectance of bands*	S2: 10 bands GF2: [B, G, R, NIR]	
Normalized difference vegetation index (NDVI)	$NDVI=\left(\rho_{NIR}-\rho_{R}\right)/\left(\rho_{NIR}+\rho_{R}\right)$	S2: [B8, B4] GF2: [NIR, R]	(Tucker, 1979)
Simple ratio index (SR)	$SR=\rho_{NIR}/\rho_{R}$	S2: [B8, B4] GF2: [NIR, R]	(Gitelson et al., 2003) (Jordan, 1969)
Normalized difference spectral index (NDSI)	$NDSI=\left(\rho_{553}-\rho_{518}\right)/\left(\rho_{553}+\rho_{518}\right)$	S2: [B3, B2]	(Patil et al., 2007)
Normalized difference water index (NDWI)	$NDWI=\left(\rho_{865}-\rho_{1614}\right)/\left(\rho_{865}+\rho_{1614}\right)$	S2: [B8A, B11]	(Gao, 1996)
Canopy chlorophyll concentration index (CCCI)	$NDFR=\left(\rho_{790}-\rho_{720}\right)/\left(\rho_{790}+\rho_{720}\right)$ $NDFR_{max}=0.576\times NDVI-0.0085$ $NDFR_{min}=0.281\times NDVI+0.0225$ $CCCI=\frac{NDFR-NDFR_{min}}{NDFR_{max}-NDFR_{min}}$	S2: [B4, B8, B8A]	(El-Shikha et al., 2007)
Enhanced vegetation index (EVI)	$EVI=\frac{2.5\left(\rho_{NIR}-\rho_{R}\right)}{\left(\rho_{NIR}+6\rho_{R}-7.5\rho_{B}+1\right)}$	S2: [B2, B4, B8] GF2: [B, R, NIR]	(Huete et al., 2002)
Spectral diversity metrics			
Coefficient of Variation (CV)	$CV=\;\frac{\sigma_{l}}{\mu_{l}}$	All VIs; All Bands	(Xu et al., 2022)
Convex Hull Area (CHA)	$\bar{CHA}=\frac{1}{S}\sum_{i=1}^{S}CHA\left(V_{i},\;\bar{V}\right)$	All Bands	(Gholizadeh et al., 2018)
Spectral Angle Mapper (SAM)	$\theta \;=cos^{-1}\;\left(\frac{\sum_{l=1}^{L}S_{il}{\bar{S}}_{l}}{{\left(\sum_{l=1}^{L}S_{il}^{2}\;\right)}^{\frac{1}{2}}\;{\left(\sum_{l=1}^{L}{\bar{S}}_{l}\right)}^{\frac{1}{2}}\;}\right)$	All Bands	(Kruse et al., 1993)
Texture features			
mean (Mean), homogeneity (Hom), contrast (Cont), dissimilarity (Dis), entropy (Ent), angular second moment (Asm), variance (Var), and correlation (Cor)	derived from the gray level co-occurrence matrix (GLCM)	The first PC of Bands	(Farwell et al., 2021)
Structural diversity metrics			
Rao’s quadratic entropy index (Rao’s Q)	$Rao^{\prime }s\;Q=\sum_{i=1}^{S}\sum_{j=1}^{S}d_{ij}*p_{i}*p_{j}$	CHM	(Torresani et al., 2020)

*B, G, R, and NIR represent blue, green, red, and NIR bands of GF2. S2 represents Sentinel-2. S2 contains 10 bands, GF2 contains 4 bands. NDFR, represents normalized difference far red index.

### 2.5 Species diversity estimation

To evaluate the impact of phenological stages and spatial resolution on forest species diversity estimation, a series of multiple stepwise regression analyses were conducted based on multi-temporal Sentinel-2 data and resampled GF2 data. To avoid overfitting caused by multicollinearity among variables, we calculated correlation coefficients (r) among all initial variables and removed highly correlated variables (r > 0.8) that had lower correlation with *in-situ* species diversity indices (**Supplementary Figure S3**-**S13**). Remaining variables were ranked based on their correlation with species diversity indices, and predictor variables were selected using forward stepwise regression. Additionally, variables with a large variance inflation factor (VIF > 10) were removed. Finally, model accuracy was assessed via leave-one-out cross-validation, using metrics of the coefficient of determination (R^2^), root mean square error (RMSE), and the mean absolute error (MAE). In terms of variable importance, hierarchical partitioning was used to separate the amount of variation explained by each predictor (Groemping, 2006).

## 3 Results

### 3.1 Impact of phenology

**Figure 3:**
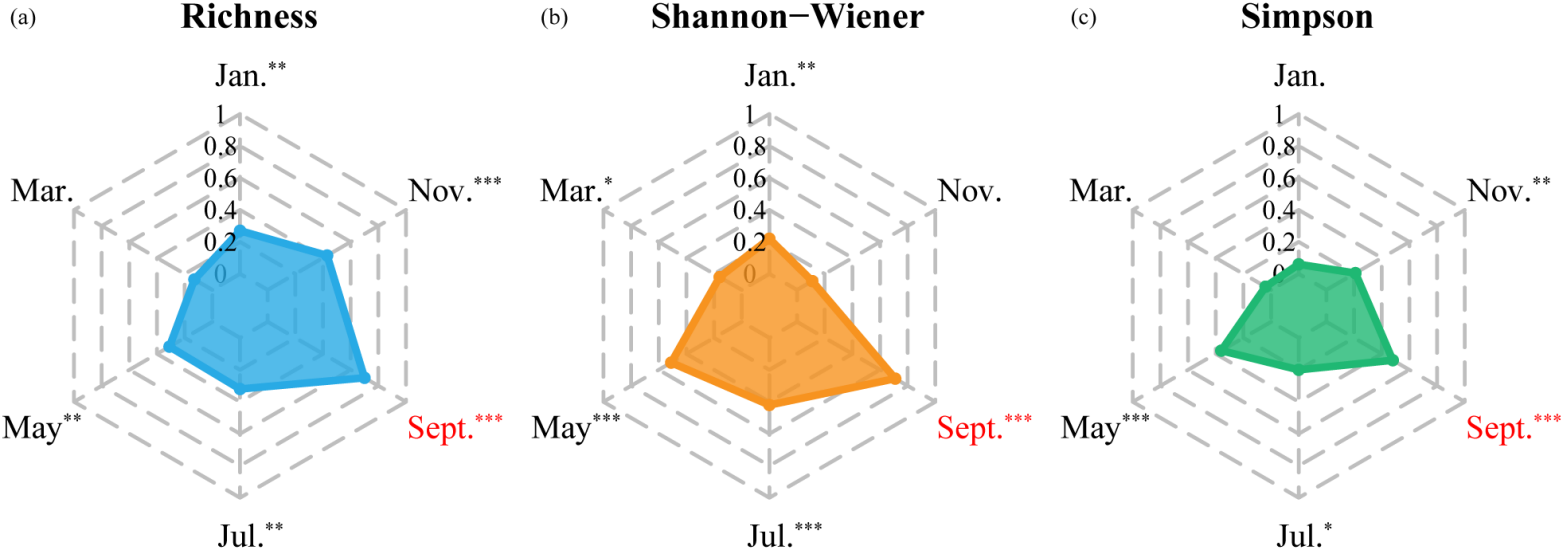
The temporal variation of coefficient of determination (R^2^) for the stepwise regression models for **(a)** Richness, **(b)** Shannon-Wiener, and **(c)** Simpson (*, 0.05< p-value <0.1, significant; **, 0.01< p-value <0.05, very significant; ***, p-value <0.01, extremely significant).

Based on best-fit models using multi-temporal Sentinel-2 data, the optimal monitoring period for Richness (R^2^ = 0.70, RMSE = 3.39, MAE = 2.49), Shannon-Wiener (R^2^ = 0.71, RMSE = 0.32, MAE = 0.26), and Simpson (R^2^ = 0.48, RMSE = 0.11, MAE = 0.09) consistently occurred in September (**Table 3**; **Figure 3**). Moreover, the model performance of Richness and Shannon-Wiener was notably superior to that of Simpson (**Supplementary Figure S2**).

**Table 3:** Model accuracies from multi-temporal Sentinel-2 images for Richness, Shannon-Wiener, and Simpson.

Model	Richness				Shannon-Wiener				Simpson			
	R^2^	RMSE	MAE	P	R^2^	RMSE	MAE	P	R^2^	RMSE	MAE	P
Jan.	0.27	5.26	4.42	0.018	0.22	0.53	0.42	0.038	0.06	0.14	0.11	0.304
Mar.	0.13	5.90	4.77	0.116	0.16	0.54	0.42	0.081	0.04	0.14	0.10	0.400
May	0.31	4.98	4.14	0.011	0.51	0.42	0.35	<0.001	0.36	0.12	0.10	0.005
Jul.	0.32	5.74	4.56	0.012	0.42	0.51	0.44	0.003	0.20	0.17	0.14	0.055
Sept.	**0.70**	3.39	2.49	<0.001	**0.71**	0.32	0.26	<0.001	**0.48**	0.11	0.09	0.001
Nov.	0.43	4.50	3.62	0.002	0.11	0.56	0.44	0.161	0.21	0.13	0.09	0.043

Bold values indicate the best model accuracies for the three species diversity indices.

### 3.2 Impact of spatial resolution

**Figure 4:**
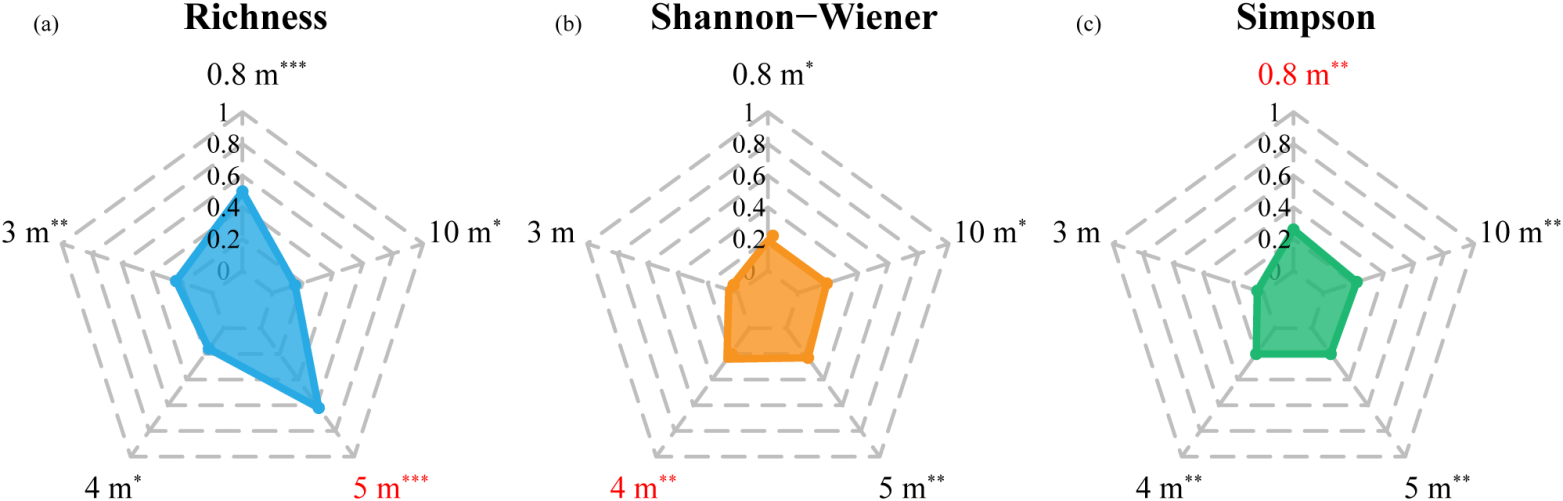
The spatial variation of coefficient of determination (R^2^) for the stepwise regression models for **(a)** Richness, **(b)** Shannon-Wiener, and **(c)** Simpson (*, 0.05< p-value <0.1, significant; **, 0.01< p-value <0.05, very significant; ***, p-value <0.01, extremely significant).

The cross-validation accuracies of the models for estimating species diversity at different spatial resolutions are shown in **Table 4** and **Figure 4**. GF2 images were more advantageous for accurate estimation of Richness compared to Simpson and Shannon-Wiener. The species richness estimation model based on 5 m resolution GF2 data performed the best among all spatial resolution scenarios (R^2^ = 0.62, RMSE = 4.11, MAE = 3.06). The accuracy for Shannon-Wiener estimation was highest at the resolution of 4 m (R^2^ = 0.24, RMSE = 0.53, MAE = 0.39), while the Simpson estimation model performed best at 0.8 m (R^2^ = 0.26, RMSE = 0.12, MAE = 0.10).

**Table 4:** Model accuracies from multi-spatial resolution GF2 images for Richness, Shannon-Wiener, and Simpson.

Model	Richness				Shannon-Wiener				Simpson			
	R^2^	RMSE	MAE	P	R^2^	RMSE	MAE	P	R^2^	RMSE	MAE	P
0.8 m	0.50	4.54	3.54	0.001	0.19	0.54	0.37	0.058	**0.26**	0.12	0.10	0.020
3 m	0.24	5.46	4.47	0.030	0.06	0.60	0.46	0.288	0.04	0.14	0.10	0.396
4 m	0.16	5.48	4.64	0.079	**0.24**	0.53	0.39	0.029	0.20	0.14	0.10	0.048
5 m	**0.62**	4.11	3.06	<0.001	0.23	0.56	0.44	0.031	0.20	0.13	0.09	0.049
10 m	0.15	5.49	4.70	0.086	0.19	0.54	0.46	0.053	0.22	0.13	0.09	0.038

Bold values indicate the best model accuracies for the three species diversity indices.

Specifically, the model at 0.8 m for Richness showed a considerable coefficient of determination (R^2^ = 0.50) and outperformed Shannon-Wiener and Simpson. Models at fine scale (0.8 m) and approaching crown size scales (4–5 m) had similar estimation accuracy for Shannon-Wiener, as same as for Simpson. Compared to the estimation based on Sentinel-2 in January, GF2 data with 10 m resolution provided a higher R^2^ for Simpson (R^2^ = 0.22), while lower R^2^ values for Richness and Shannon-Wiener.

### 3.3 Importance of heterogeneity metrics

**Figure 5:**
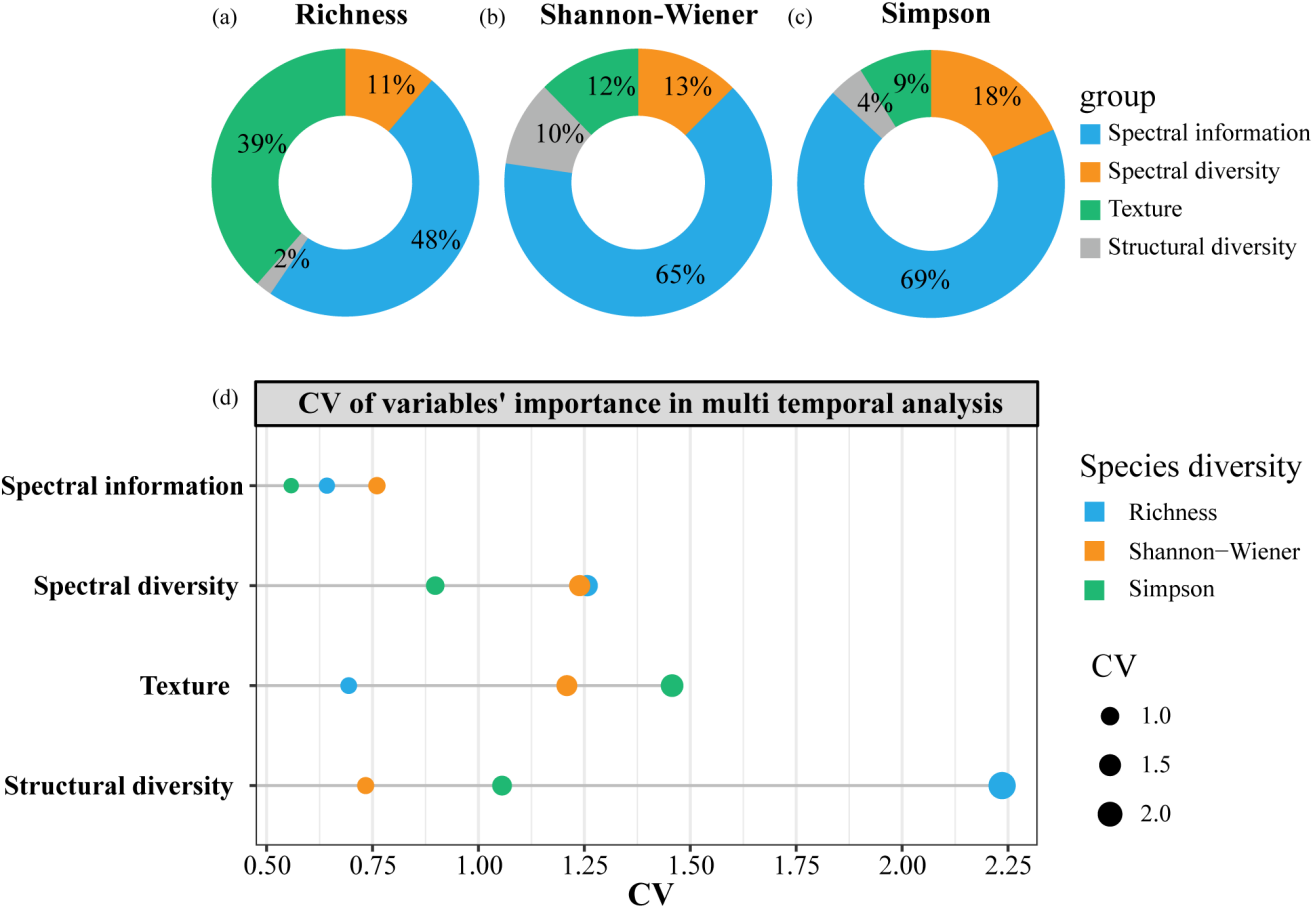
The overall explanatory power of four types of heterogeneity metrics on species diversity indices in phenological effect analysis **(a-c)**; and the explained variance’s CV of four types of heterogeneity metrics in different temporal models **(d)**.

In phenological effect analysis, spectral information metrics exhibited the highest explanatory power, accounting for 48% of variance in Richness and over 60% for Shannon-Wiener and Simpson. Texture features explained more variance in Richness (39%) than in Shannon-Wiener (12%) and Simpson (9%). Spectral diversity metrics explained 11%, 13%, and 18% of the variance for Richness, Shannon-Wiener, and Simpson, respectively. Structural diversity metrics had the lowest explanatory power, contributing 10% for Shannon-Wiener, 4% for Simpson, and 2% for Richness (**Figure 5a–c**). Additionally, the temporal variability of variable contributions (**Figure 5d**) indicated that the explanatory power of spectral information remained relatively stable across all phenological periods. However, the metric with the greatest variation in importance differed depending on diversity indices. For Richness, the structural diversity metric showed the greatest temporal variation in importance, while it was the spectral diversity metric for Shannon-Wiener, and the texture feature for Simpson.

**Figure 6:**
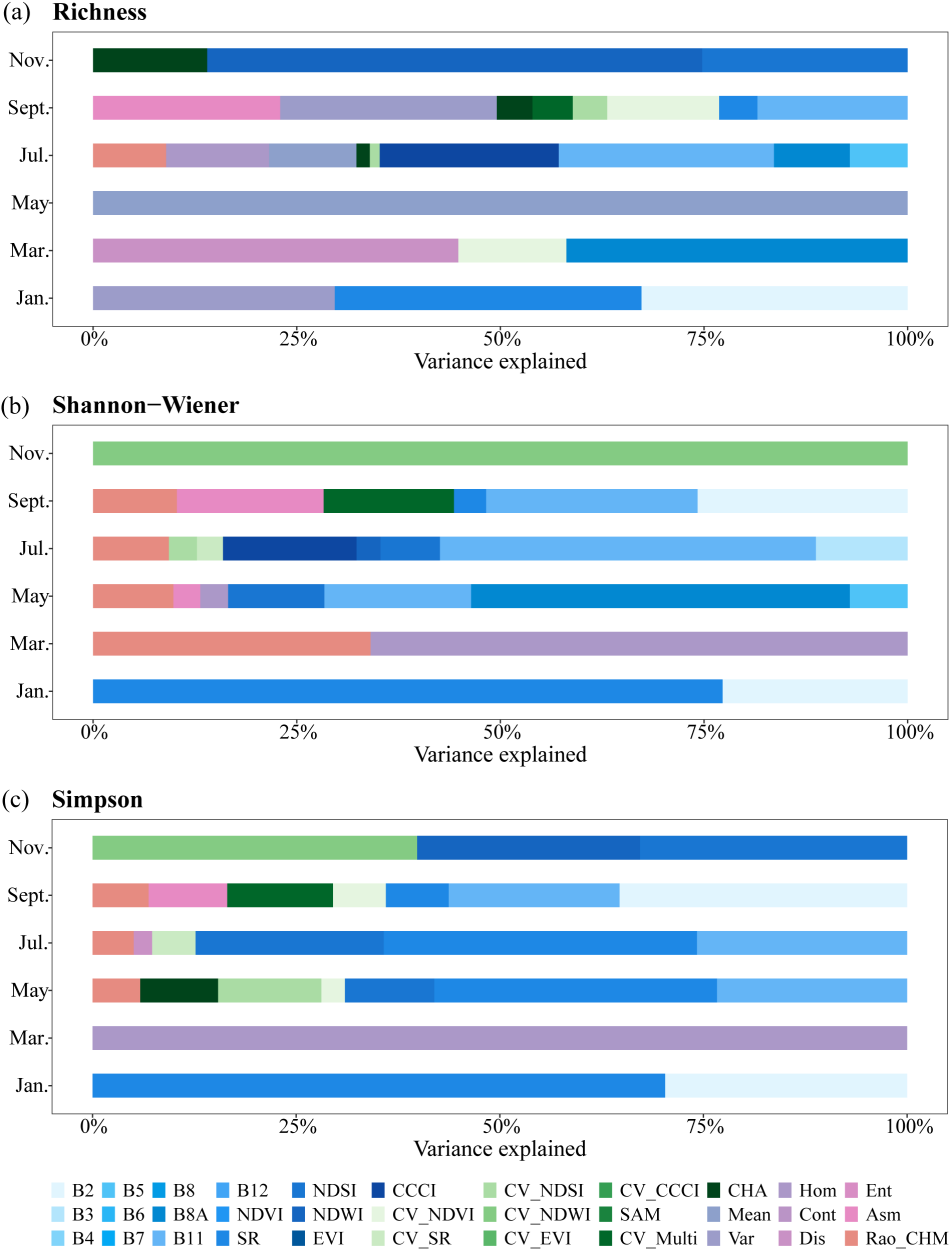
Hierarchical partitioning of the variation explained for **(a)** Richness, **(b)** Shannon-Wiener, and **(c)** Simpson in different phenological models.

The hierarchical partitioning of the variation explained by each variable for species diversity models at different phenological stages is shown in **Figure 6**. Specifically, B2 and SR played important roles in the January models for all species diversity indices (33% and 38% for Richness, 23% and 77% for Shannon-Wiener, 30% and 70% for Simpson). B11 explained variation in Shannon-Wiener and Simpson models from May to September, and contributed to Richness models in July (26%) and September (18%). Additionally, NDWI and NDSI were involved in Richness estimation model at November, while acted on Shannon-Wiener models in May and July, and contributed to Simpson models across these three periods (May, July, and November). Moreover, the texture feature of Mean independently explained all variance in Richness in the May model, CV_NDWI accounted for all variance in Shannon-Wiener in the November model, and Hom explained all variance in Simpson in the March model.

**Figure 7:**
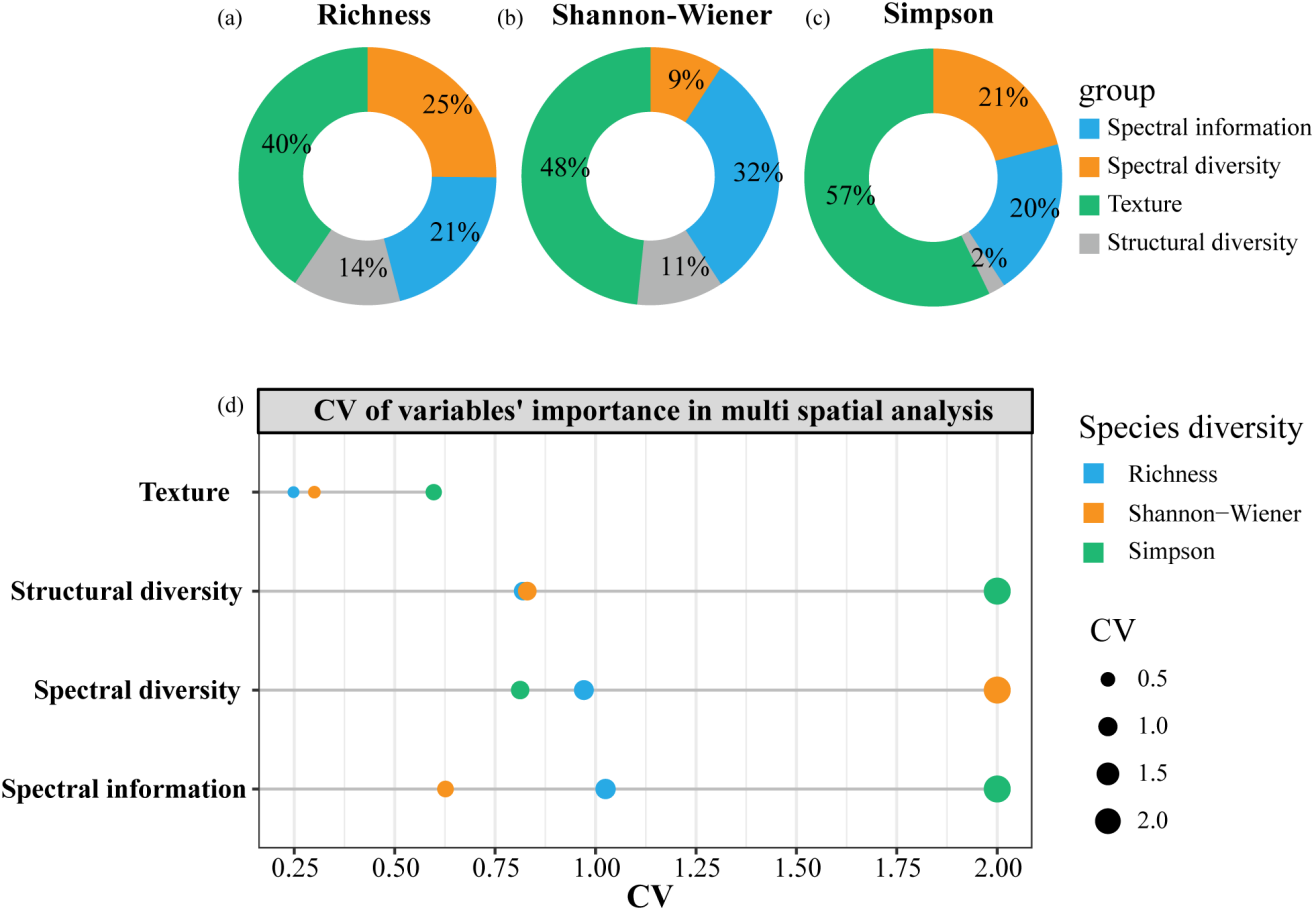
The overall explanatory power of four types of heterogeneity metrics on species diversity indices in spatial scale effect analysis **(a-c)**; and the explained variance’s CV of four types of heterogeneity metrics in different spatial models **(d)**.

**Figure 7a–c** showed significant differences in the importance of four types of heterogeneity metrics for species diversity estimation at different spatial scales. Overall, texture features were the most important, explaining 40%, 48%, and 57% of the variance in Richness, Shannon-Wiener, and Simpson index, respectively. Spectral information contributed 21% to Richness, 32% to Shannon-Wiener, and 20% to Simpson. Spectral diversity performed better for Richness (25%) and Simpson (21%) than Shannon-Wiener (9%). Structural diversity showed stronger explanatory power for Richness (14%) and Shannon-Wiener (11%) than Simpson (2%). Moreover, the contribution of texture features remained stable across scales for all three diversity indices (**Figure 7d**). Spectral diversity varied most in contribution for Shannon-Wiener estimation across different spatial resolutions, while spectral information and structural diversity varied significantly in importance for Simpson estimation across scales.

**Figure 8:**
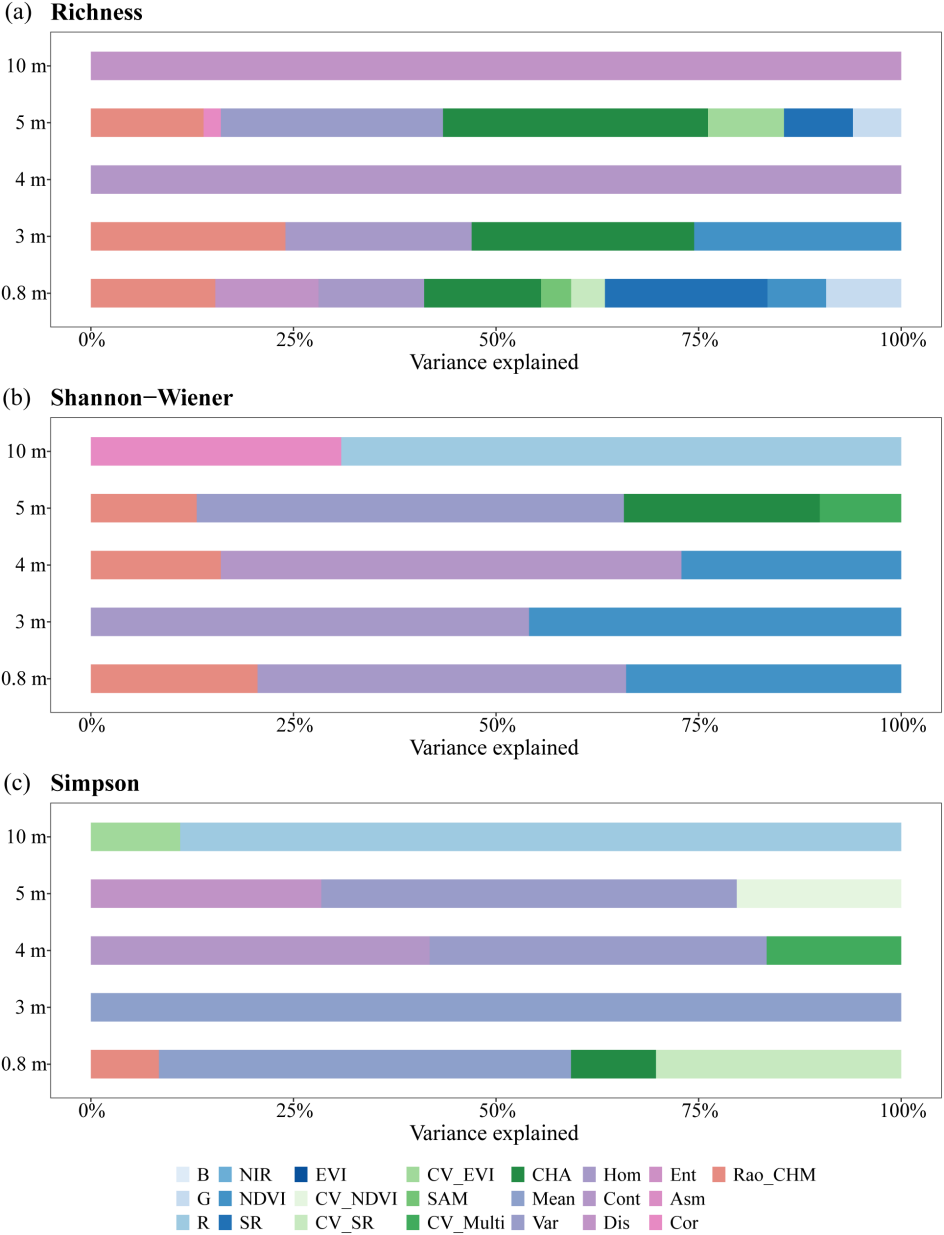
Hierarchical partitioning of the variation explained for **(a)** Richness, **(b)** Shannon-Wiener, and **(c)** Simpson in different spatial models.

As shown in **Figure 8**, texture features Dis, Cont, and Mean completely explained Richness at 10 m and 4 m, and Simpson at 3 m. NDVI was the most important spectral information variable for Shannon-Wiener, contributing significantly at 0.8 m, 3 m and 4 m resolutions (34%, 46%, and 27%). CHA contributed to Richness estimation at three scales (0.8 m: 14%, 3 m: 27%, and 5 m: 33%), while only one scale for Shannon-Wiener (24% at 5 m) and Simpson (10% at 0.8 m). Rao’s Q based on CHM was involved in three spatial models for Richness (0.8 m, 3 m, and 5 m), and Shannon-Wiener (0.8 m, 4 m, and 5 m), but only one model for Simpson (0.8 m).

## 4 Discussion

### 4.1 Phenological sensitivity of species diversity estimation

We identified September as the optimal phenological period, which aligns with the consensus that the growing season (May–September) is the most informative period and a primary choice for remote sensing-based diversity monitoring. However, inconsistencies exist in literature regarding the most efficient single-date time window for estimating forest species diversity. While many studies underscored the importance of transitional seasons, such as green-up (May) and senescence (October) (Liu et al., 2023a), others suggested that peak growing seasons like July can obtain higher accuracy (Arekhi et al., 2017; Chrysafis et al., 2020; Yang et al., 2022). These differences stem from the biochemical and morphological changes among species caused by crucial phenological events, such as leaf unfolding and flowering in spring, and leaf discoloration and falling in autumn (Kollert et al., 2021; Xi et al., 2023). In our tropical site, some tree species begin fruiting in September, leading to the transformation of dominant pigments in leaves and driving color changes from green to yellow and red, which enhances tree species differentiation (Kapoor et al., 2022). Although the effect of shrubs and grasses was relatively low under dense canopy cover in tropical forests, seasonal variations in non-tree vegetation or background signals may also affect the relationship between spectral heterogeneity and species diversity (Madonsela et al., 2021), potentially explaining the reduced accuracy in November. The phenological asynchrony in different forest layers responding to warming temperatures in May may increase the spectral contribution of non-canopy vegetation, further reducing accuracy. These findings underscore the importance of understory vegetation in species diversity assessments, which may be better captured in the future with advancements in sensor technologies, such as hyperspectral LiDAR (Bai et al., 2024) and multi-angle observations and satellite thermal infrared sensing (Adams et al., 2025).

Our results highlighted that late spring and autumn are critical temporal windows for forest species diversity monitoring. However, frequent cloud cover and rainfall in tropical regions increase the uncertainty in acquiring images during a single optimal period. Moreover, effective phenological time for diversity monitoring varies by vegetation type. For example, broadleaved tree species are typically more distinguishable in autumn, while conifers exhibit greater spectral changes in spring (Grabska et al., 2019). Hence, if September imagery is unavailable, May or November images can also be considered based on vegetation characteristics. We acknowledge that bimonthly temporal selection may be insufficient to fully capture subtle phenological changes in tropical evergreen vegetation. New generation satellites with higher spatiotemporal resolution are expected to address this limitation. For instance, the PlanetScope dataset characterized by daily revisit frequency and 3 m spatial resolution exhibits considerable potential to comprehensively assess forest diversity. Besides, continuous advancements in the fusion of multi-source remote sensing data offer promising opportunities for more effective monitoring of tropical forests at both high temporal frequency and fine spatial resolution. Notably, Multi-Temporal and Spectral-Temporal-Metric methods are increasingly applied to forest species diversity mapping (Rahmanian et al., 2023; Vanguri et al., 2024). We recommend performing a similar phenological sensitivity analysis before image composition to reduce data redundancy and improve accuracy.

### 4.2 Scale dependency of species diversity estimation

Our results indicated that the optimal spatial resolution varied among species diversity indices. The model accuracy of Richness and Shannon-Wiener was highest when the spatial resolution approaches the average crown size (4–5 m). This is because, at this resolution, the spectral properties effectively capture the chemical, morphological, and structural characteristics of individual trees (Zheng et al., 2022). However, Simpson performed better at finer resolutions (0.8 m), likely due to its sensitivity to dominant species. Considering the overall low abundance of dominant species caused by the high diversity in this study area, at resolutions of 5 m or alike, the spectral information of the coniferous dominant species is easily concealed by that of broadleaved species, while it could be preserved at fine resolution. Although finer resolutions may introduce noise, appropriate data preprocessing effectively mitigates this risk, ensuring reliable results. Another point worth noting is the number of *in-situ* plots, which may not fully represent the community composition and distribution in the study area. Despite our best efforts to sample as evenly as possible, the limited *in-situ* plots with high abundance of dominant species may impact the results.

Some previous studies have compared the impact of spatial scale on species diversity estimation using different satellite images with multiple resolutions (Gyamfi-Ampadu et al., 2021). However, differences in spectral properties of satellite data sources may cause bias in evaluating scale effects. To address this, we employed a single type of satellite data and resampling method, focusing on identifying suitable monitoring scales to provide informed recommendations. Moreover, evidence shows that resampling high-resolution imagery to coarser resolutions may improve the estimation accuracy up to a threshold, beyond which spectral mixing reduces performance (Liu et al., 2024). Additionally, a coarser resolution resulted in fewer pixels available within the plot for calculating the diversity indices, partly explaining the lower accuracy observed at 10 m resolution in our study. Beyond the critical spatial scales tested in our study, we suggest future research paying more attention to the integrated utilization of nested plots and UAV data to better understand the effects of grain and extent on forest species diversity estimation. A critical next step is to assess how spatial resolution degradation influence spectral integrity and metric performance, with UAV imagery providing a reliable ground reference. UAV provides very high-resolution data, which can match well with the nested plots at different scales, thereby enabling spatial extrapolation and selecting the optimal monitoring scale. Adjusting the field sampling design to be remotely driven may be also a viable strategy to probe deeply into scale effects. Besides, while this study was targeted at tropical forests, the methodology for scale detection is broadly applicable to other ecosystems. Notably, in areas with large terrain undulations, such as mountainous and canyon areas, terrain correction and shadow factors need to be carefully taken into consideration.

### 4.3 Variable importance in species diversity estimation

Our results indicated that the importance of spectral heterogeneity metrics in forest species diversity estimation has temporal and spatial effects. In our study, the blue band was prominent in January, probably associated with carotenoid uptake during leaf senescence (Hennessy et al., 2020). The near-infrared and red-edge bands were significant in late spring, attributed to their association with strong chlorophyll uptake and internal leaf structure during early growth stages. The SWIR band contributed to monitoring species diversity across several phenological periods, potentially due to its sensitivity to canopy leaf water content (Ming et al., 2024). However, for GF2, we only found a weak role of Green, Red, and NDVI, which may be related to the sensor band range and the limited number of bands. But interestingly, when resampling to 10 m resolution, the spectral band mattered more significantly to Simpson and Shannon-Wiener estimation compared to spectral diversity indices that integrate multidimensional information. This phenomenon, where more complex metrics using full-range spectral data perform worse on coarse-resolution images, has also been reported (Rossi et al., 2021; Wang et al., 2018b). In addition, spectral diversity metrics calculated by Sentinel-2 showed limited explanatory power for species diversity, while CHA based on GF2 explained a larger proportion of the variance. This discrepancy may be attributed to the coarse spatial resolution of Sentinel-2 and its matching issue with the plot size. Previous studies also reported that the relationship between species and spectral diversity is more significant at larger plot size (Marselis et al., 2019). More refined spatial matching strategies, such as increasing field plot size, applying sampling buffers or comparing the performance of satellite data extracted from different corner coordinates (Liu et al., 2023b), could contribute to mitigate the effects of positional offset. In addition, different atmospheric correction methods may affect metric consistency across time and space (Chraibi et al., 2022). While the use of normalized vegetation indices (VIs) can mitigate some atmospheric effects, band reflectance values used in spectral diversity metrics remain sensitive to correction accuracy. Given the complexity of atmospheric conditions in tropical regions, future research could investigate the consistency and uncertainty of different atmospheric correction methods, and its potential influence on spectral diversity estimation. Also, the scale aggregation led to various degrees of spectral mixing, which mitigated the importance of spectral heterogeneity metrics. Therefore, the availability of more satellite-based data with high temporal and spatial resolution or multi-temporal UAV data is called for further exploring the suitability of spectral heterogeneity metrics at different spatial and temporal scales.

Our analysis also emphasized the critical role of structural heterogeneity metrics, especially texture features, in forest species diversity estimation. These features derived from the spatial variability in image pixels can effectively reflect variations in canopy structure, leaf arrangement, and key aspects of ecosystem heterogeneity that influence forest diversity patterns. Compared to other metrics, the superior performance of texture features likely benefits from the fact that they can be computed through diverse algorithms, enabling the quantification of multidimensional image heterogeneity (Farwell et al., 2021). With the emergence of more high-precision data, texture features offer promising potential to enhance species diversity prediction models. The limited structural diversity metrics (Rao’s Q and texture) in our study may underestimate the importance of structural metrics for species diversity estimation. Thus, we suggest fully leveraging the advantages of LiDAR point cloud data and incorporating innovative structural heterogeneity indices, such as canopy entropy, to reduce saturation effects in structurally complex forests, and improve the accuracy of diversity estimation. Moreover, with the recent availability of high-resolution, wall-to-wall structural parameter datasets, such as the canopy height map at 1 m resolution (Tolan et al., 2024), the influence of structural heterogeneity can be integrated in future large-scale applications.

## 5 Conclusion

Overall, we assessed the impacts of phenology and spatial resolution on species diversity estimation in a typical tropical forest using Sentinel-2 and GF2 satellite data and emphasized the importance of various heterogeneity metrics across temporal and spatial scales. Our results indicate that the optimal phenological periods for estimating species diversity are at the beginning and end of the growing season, while the ideal spatial resolution aligns with the tree crown size. These findings provide guidance on selecting appropriate phenological periods and spatial scales, thereby improving species diversity monitoring in complex evergreen tropical forests to achieve more accurate and efficient estimations. Despite focusing on a specific tropical forest, our methodology could offer concrete spatiotemporal prior knowledge for integrating suitable remote sensing data and optimizing fieldwork designs in various ecosystems. Broader exploration across globally diverse tropical forests would enhance the understanding of tropical-specific phenological windows and spatial scales. Additionally, our study provides valuable insights into promoting the development of dynamic heterogeneity metrics that incorporate spatiotemporal variation and calling for in-depth analysis of the impact of scale changes on metrics themselves. Future research could explore the synergistic application of multi-source satellite imagery, UAV data, and machine learning algorithms, which may help bridge the scale gap between *in-situ* measurements and large-scale satellite observations, and further enhance the accuracy and scalability of species diversity monitoring.

## Data Availability

Publicly available datasets were analyzed in this study. This data can be found here: Multi-temporal Sentinel-2 data were retrieved from https://dataspace.copernicus.eu/. GF2 data was retrieved from http://www.sasclouds.com/. UAV LiDAR data was private and not publicly available, please contact the corresponding author via email if interested. Field plots data was available in the **Supplementary Material**.
