# Supplementary material for: Species diversity estimation in a typical tropical forest: which phenological stage and spatial resolution are suitable?
Source: Front Plant Sci. 2025 Aug 18;16:1582910. doi: 10.3389/fpls.2025.1582910 (PMC12399664; doi:10.3389/fpls.2025.1582910)
Supplement: Supplementary file 1 [file DataSheet1.docx]

Supplementary Material

# Supplementary Figures and Tables

## Supplementary Figures


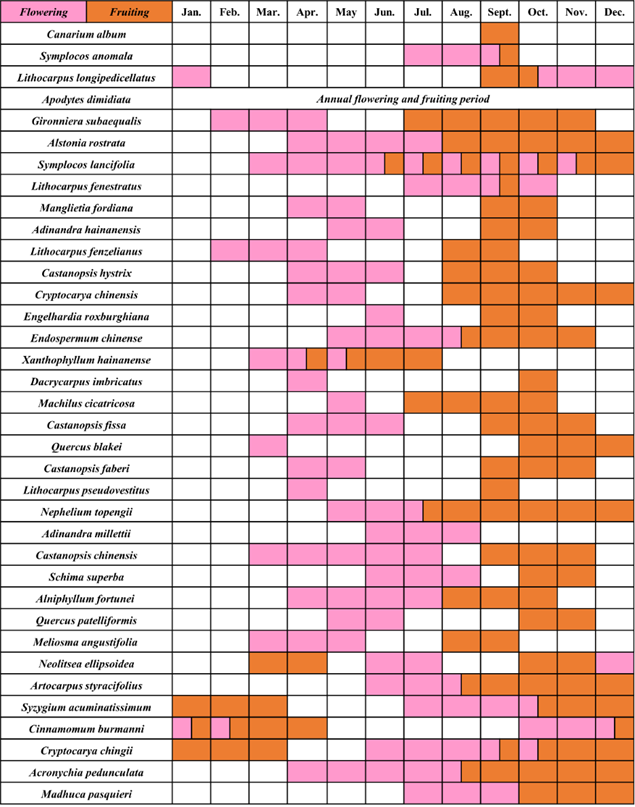


**Supplementary Figure 1.** Asynchrony in flowering and fruiting periods of the dominant trees. Among them, only *Dacrycarpus imbricatus* is coniferous tree.

**
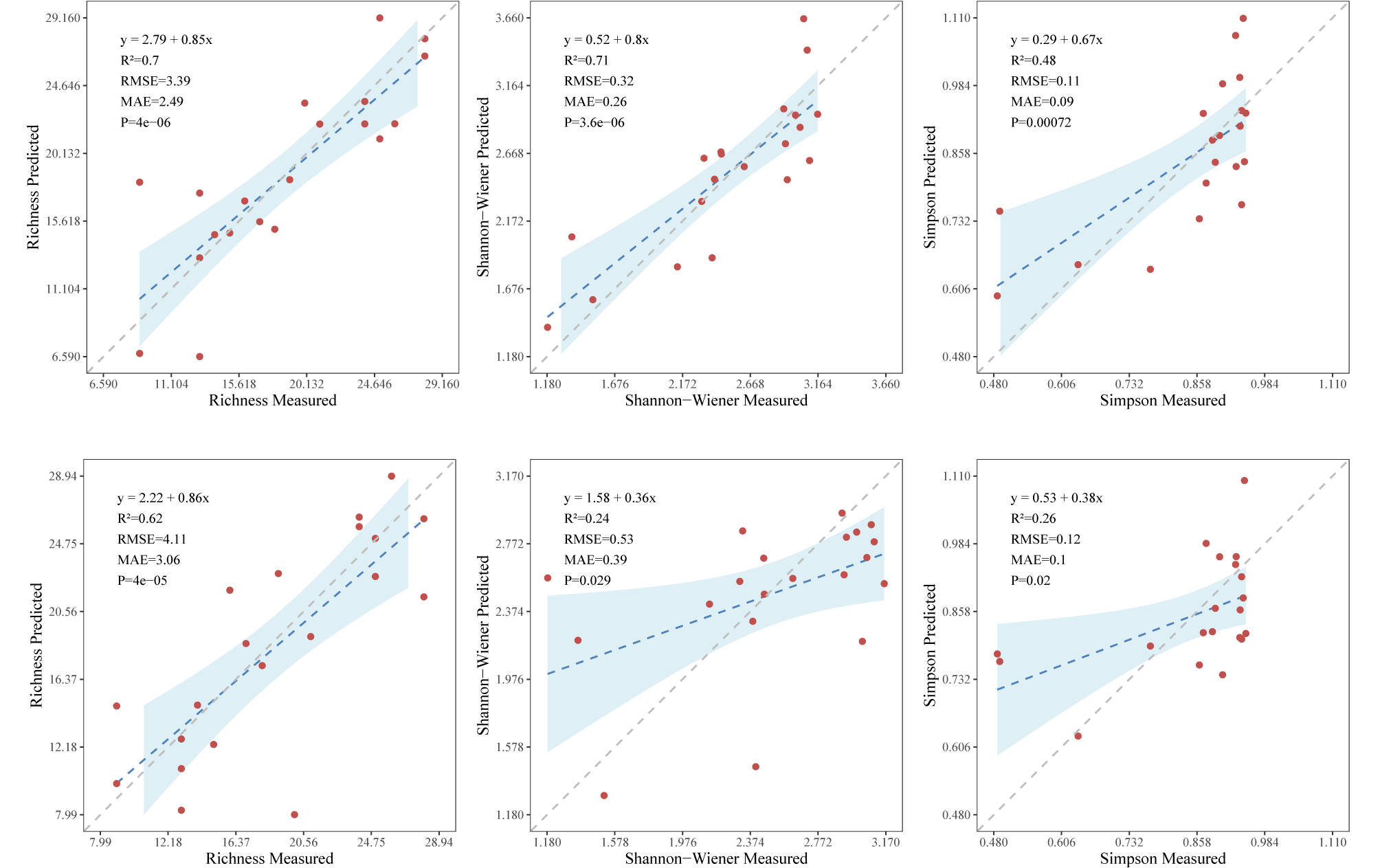
**

Supplementary Figure 2. Scatter plots of the field-measured and predicted species diversity indices at optimal phenological stage (upper) and spatial resolutions (lower). The optimal phenological stage is September for all indices. The optimal spatial resolution is 5 m for Richness, 4 m for Shannon-Wiener index, and 0.8 m for Simpson index, respectively.


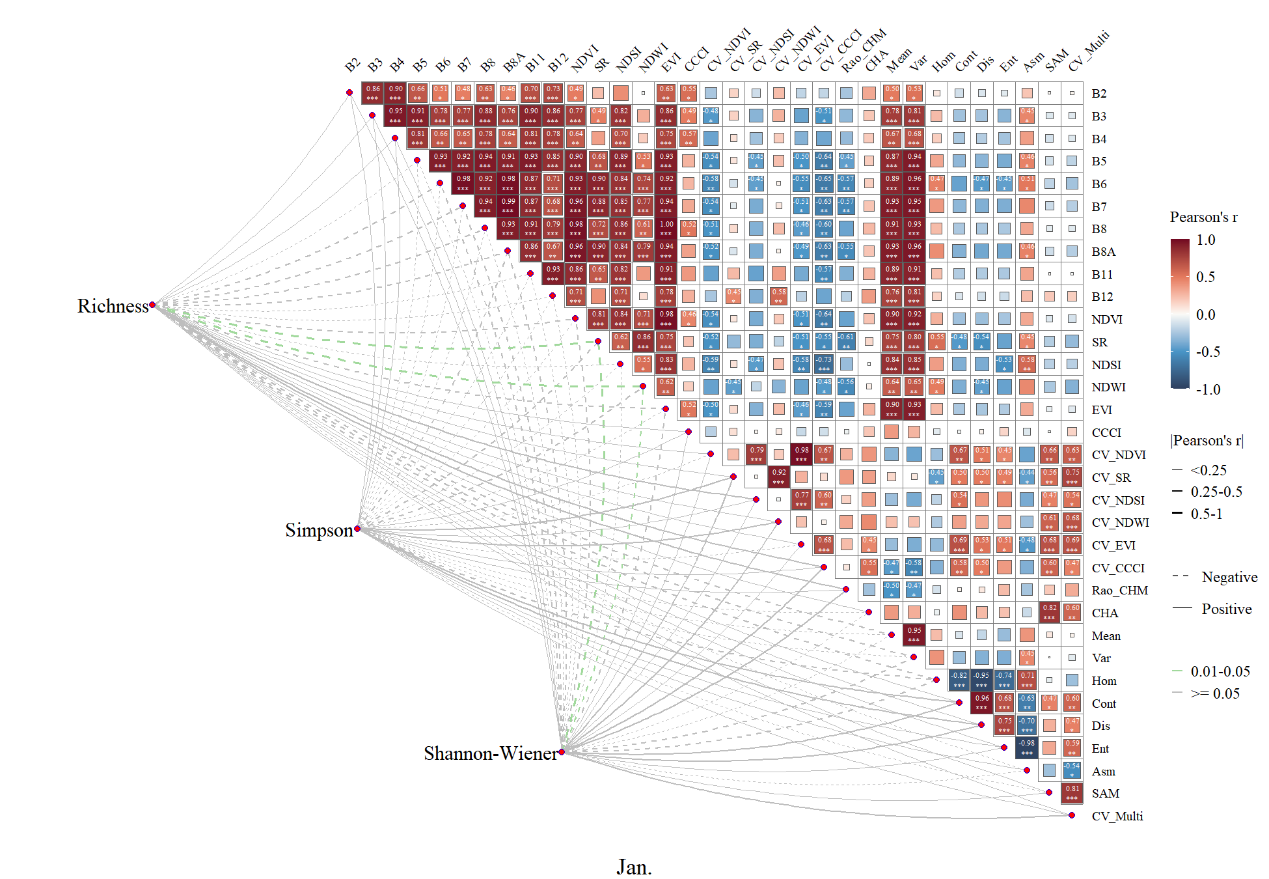


Supplementary Figure 3. Correlogram between in-situ species diversity and metrics based on Jan. image from Sentinel-2 and UAV LiDAR data.


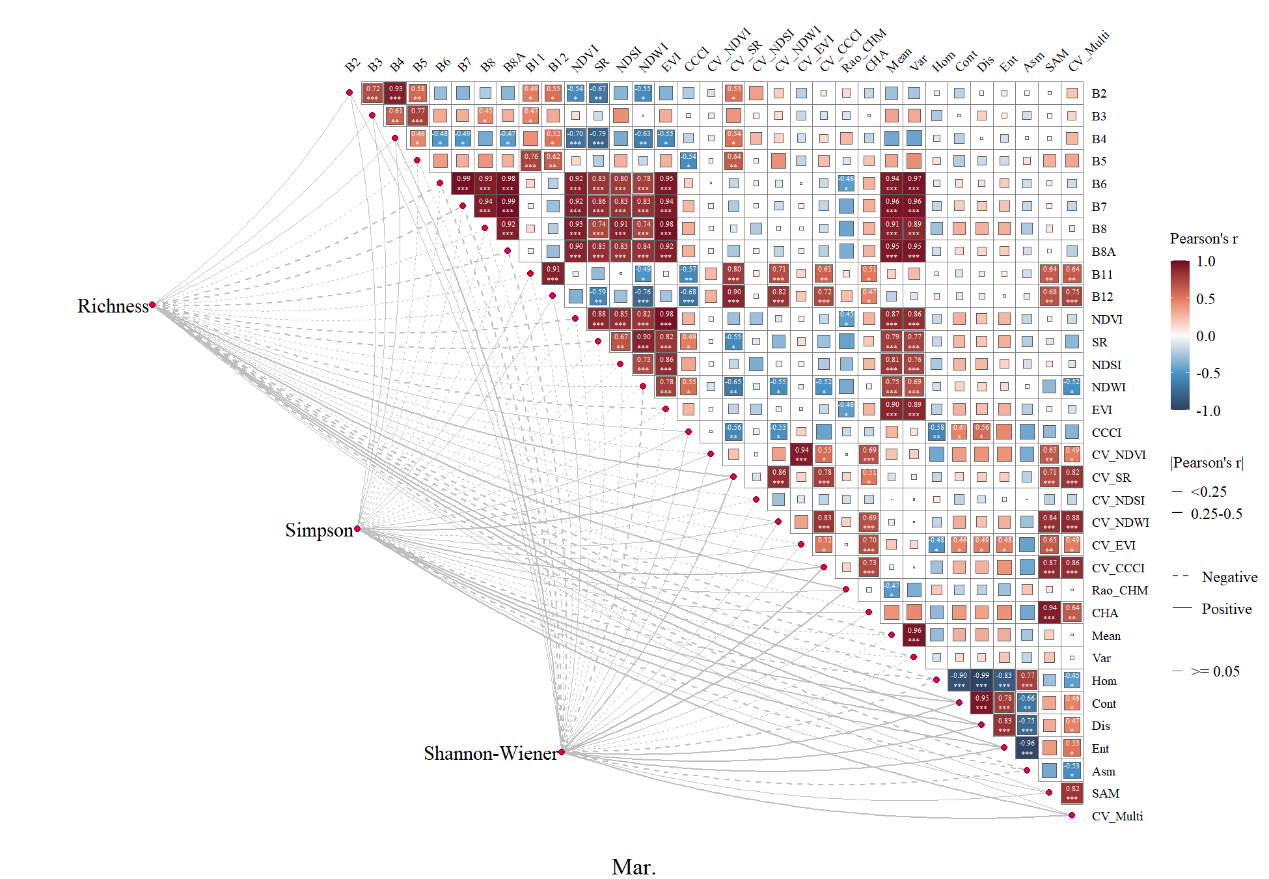


Supplementary Figure 4. Correlogram between in-situ species diversity and metrics based on Mar. image from Sentinel-2 and UAV LiDAR data.


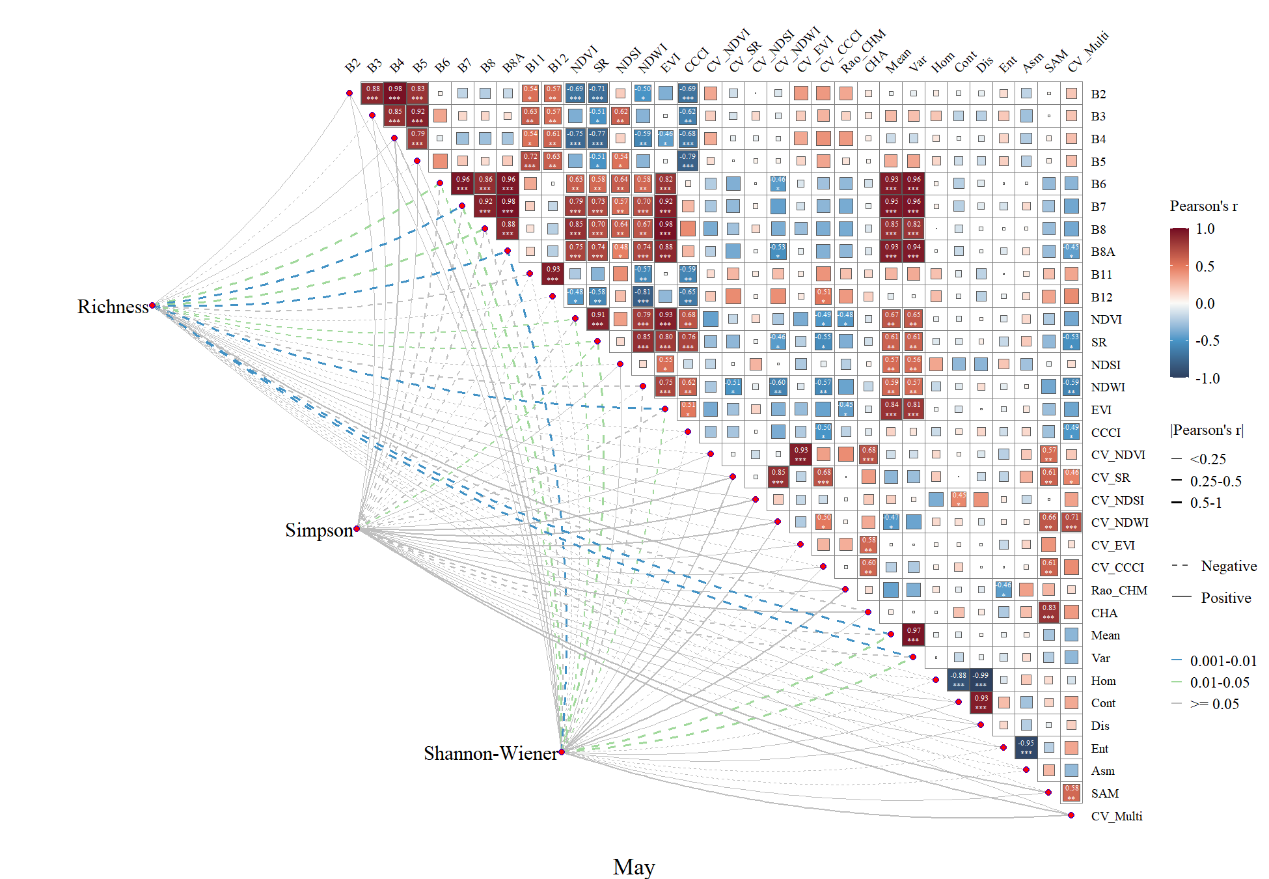


Supplementary Figure 5. Correlogram between in-situ species diversity and metrics based on May image from Sentinel-2 and UAV LiDAR data.


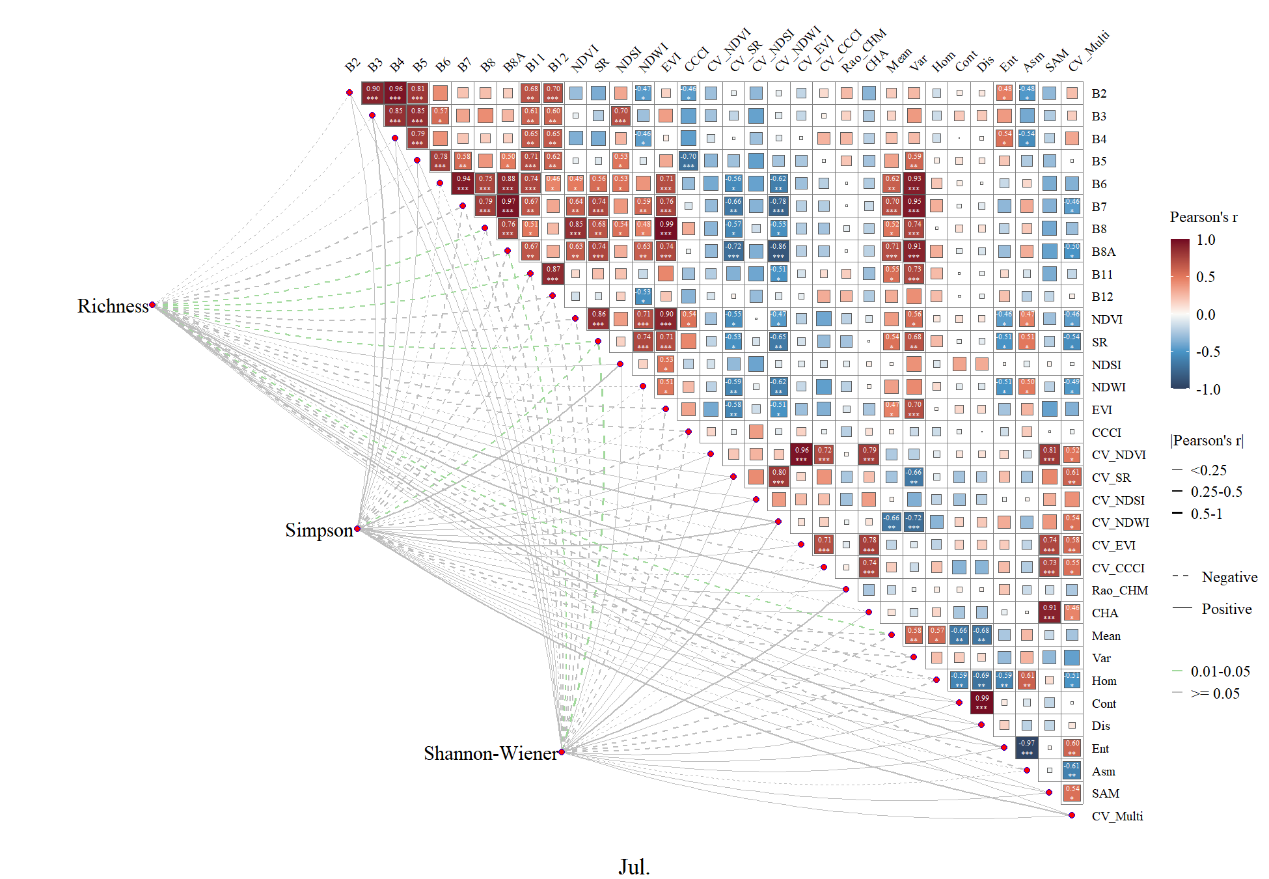


Supplementary Figure 6. Correlogram between in-situ species diversity and metrics based on Jul. image from Sentinel-2 and UAV LiDAR data.


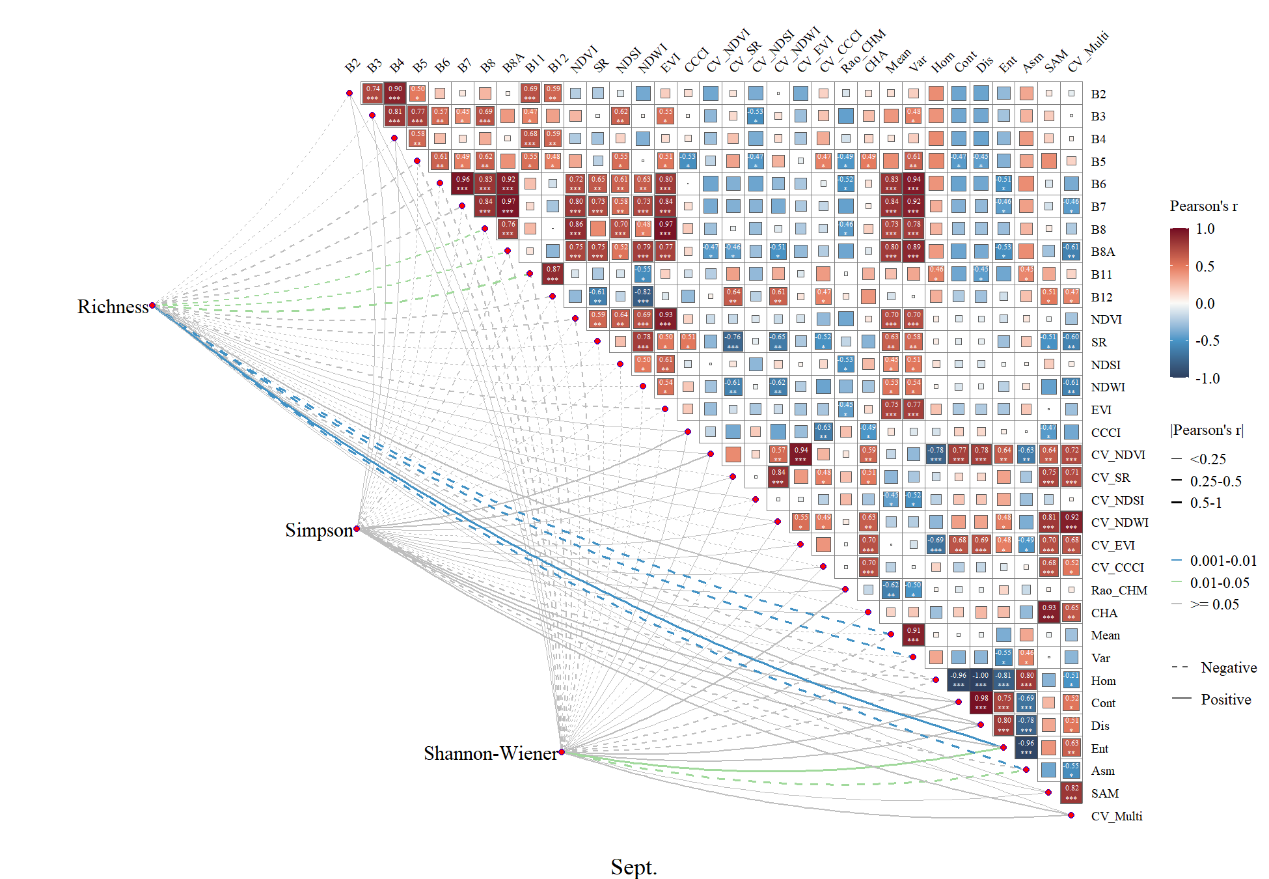


Supplementary Figure 7. Correlogram between in-situ species diversity and metrics based on Sept. image from Sentinel-2 and UAV LiDAR data.


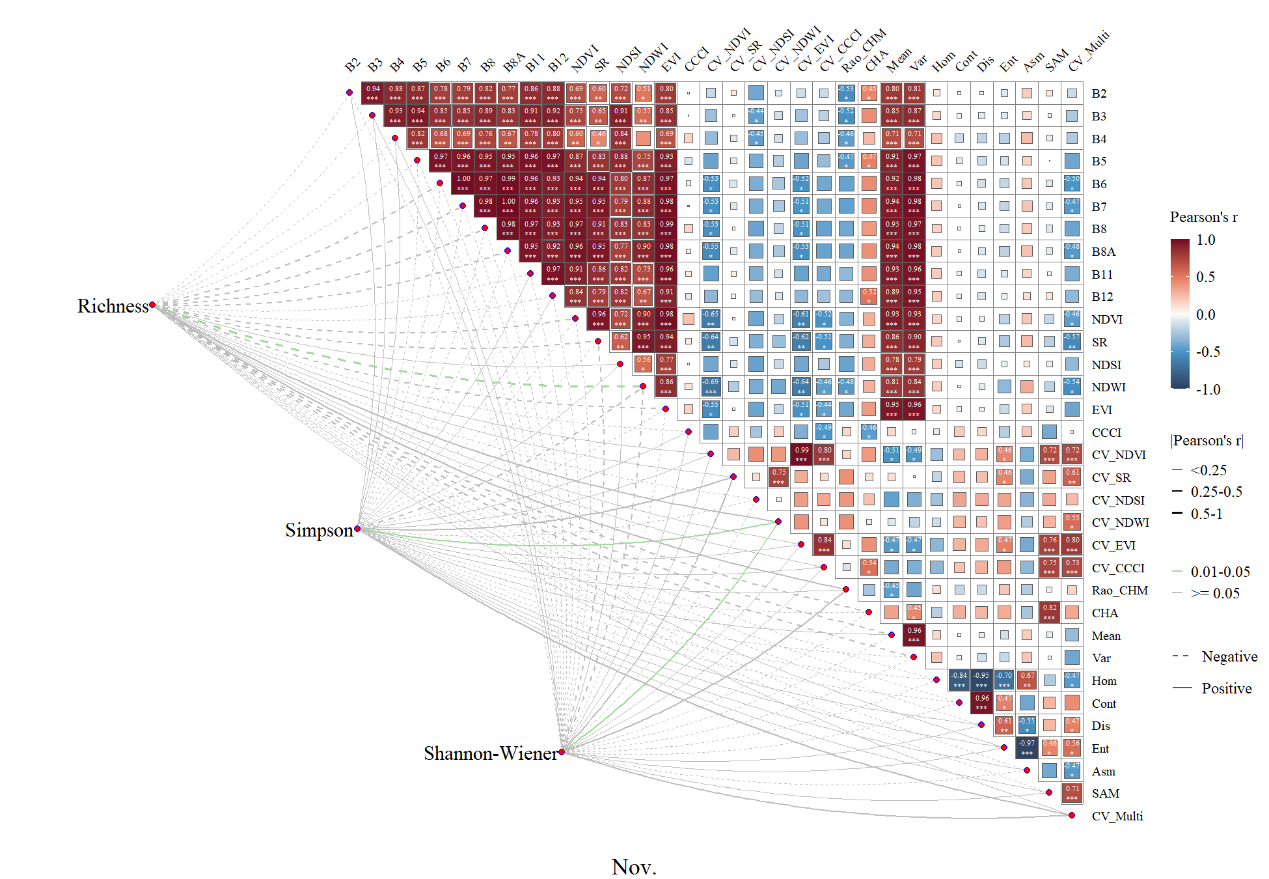


Supplementary Figure 8 Correlogram between in-situ species diversity and metrics based on Nov. image from Sentinel-2 and UAV LiDAR data.


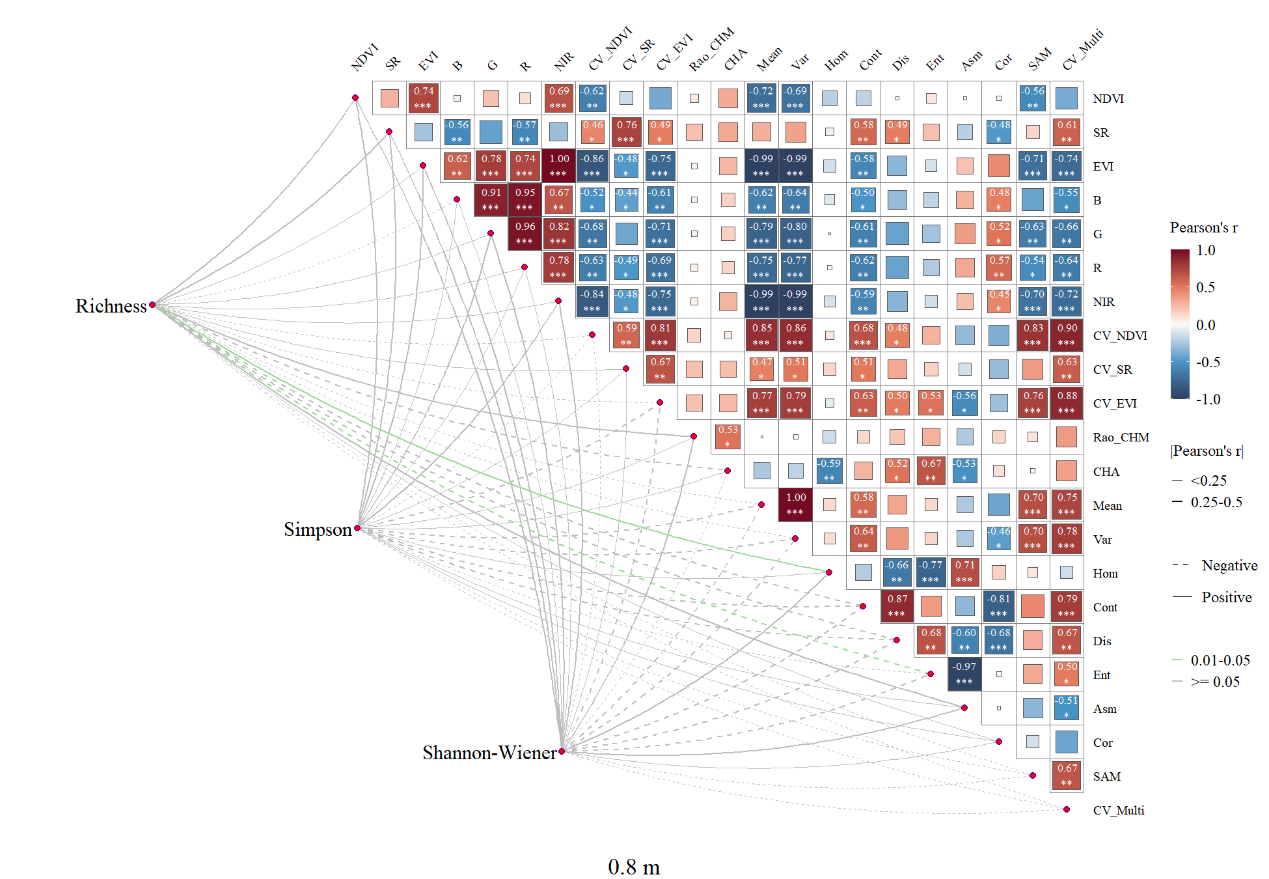


Supplementary Figure 9 Correlogram between in-situ species diversity and metrics based on 0.8 m image from GF2 and UAV LiDAR data.


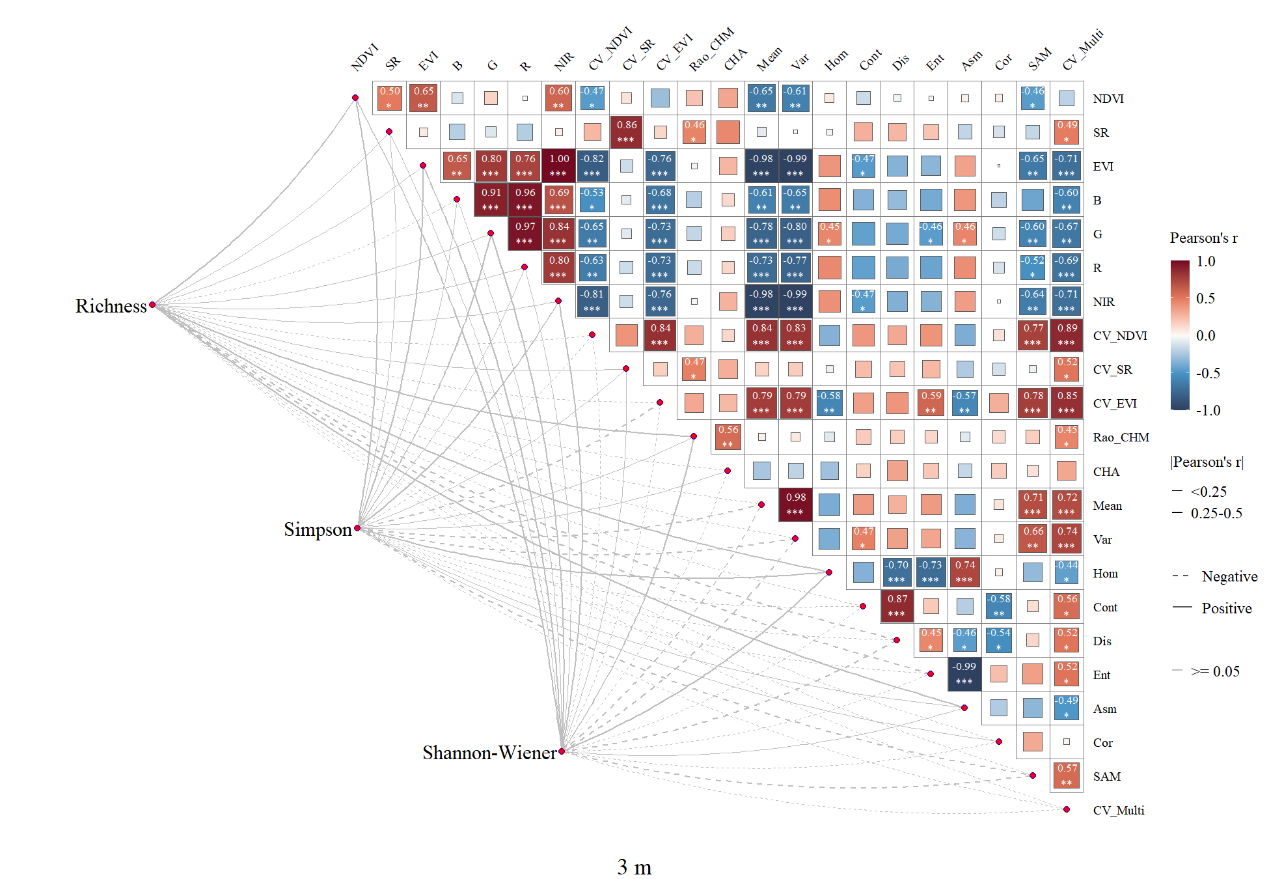


Supplementary Figure 10 Correlogram between in-situ species diversity and metrics based on 3 m image from GF2 and UAV LiDAR data.


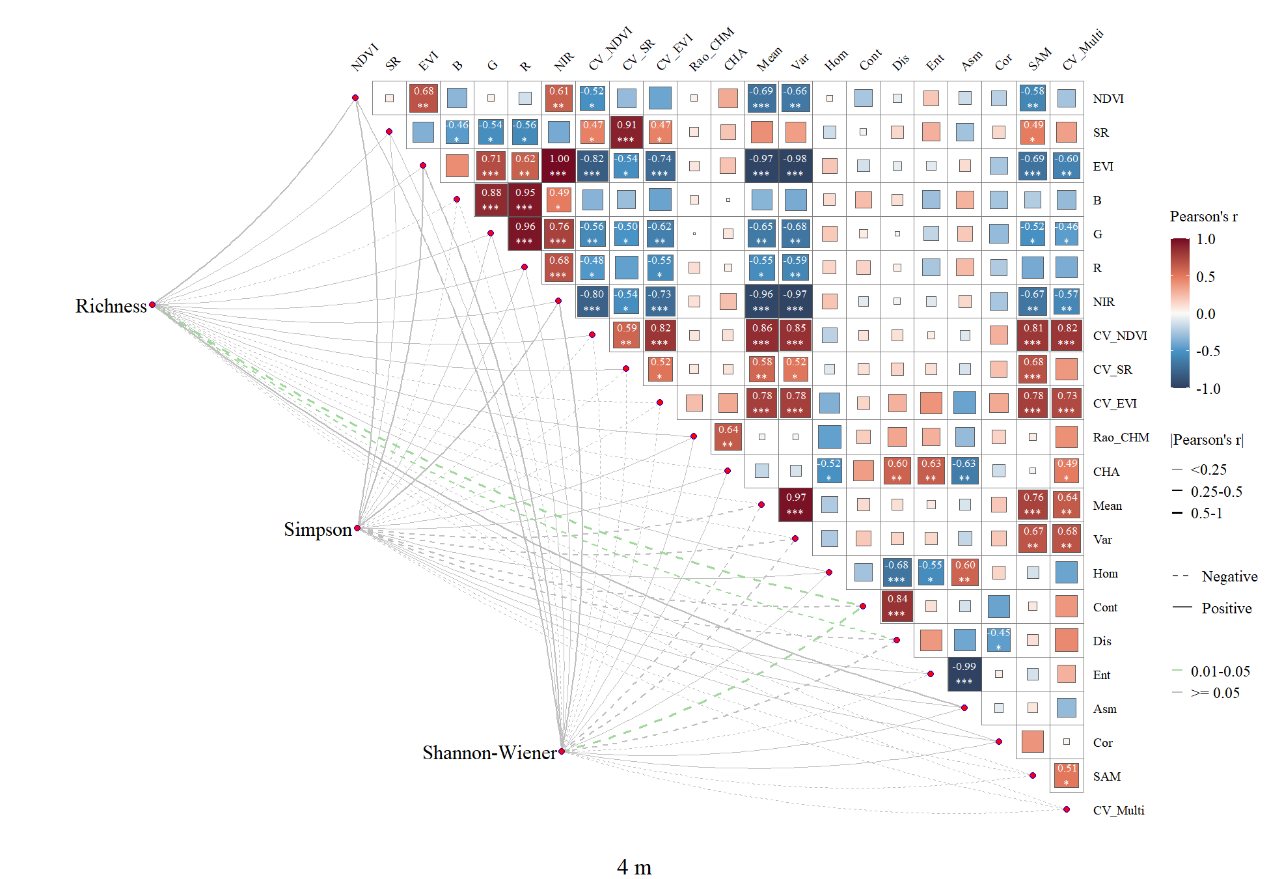


Supplementary Figure 11 Correlogram between in-situ species diversity and metrics based on 4 m image from GF2 and UAV LiDAR data.


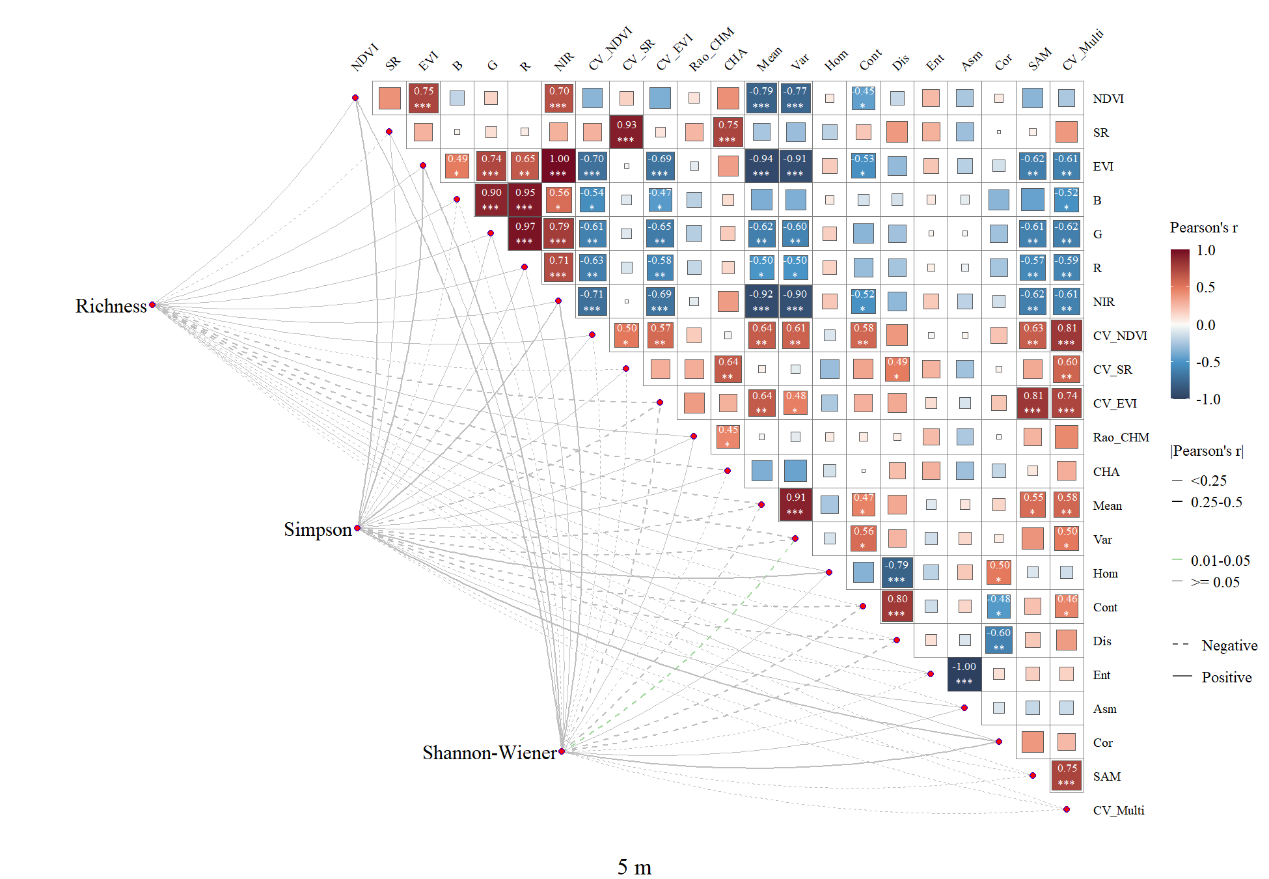


Supplementary Figure 12 Correlogram between in-situ species diversity and metrics based on 5 m image from GF2 and UAV LiDAR data.


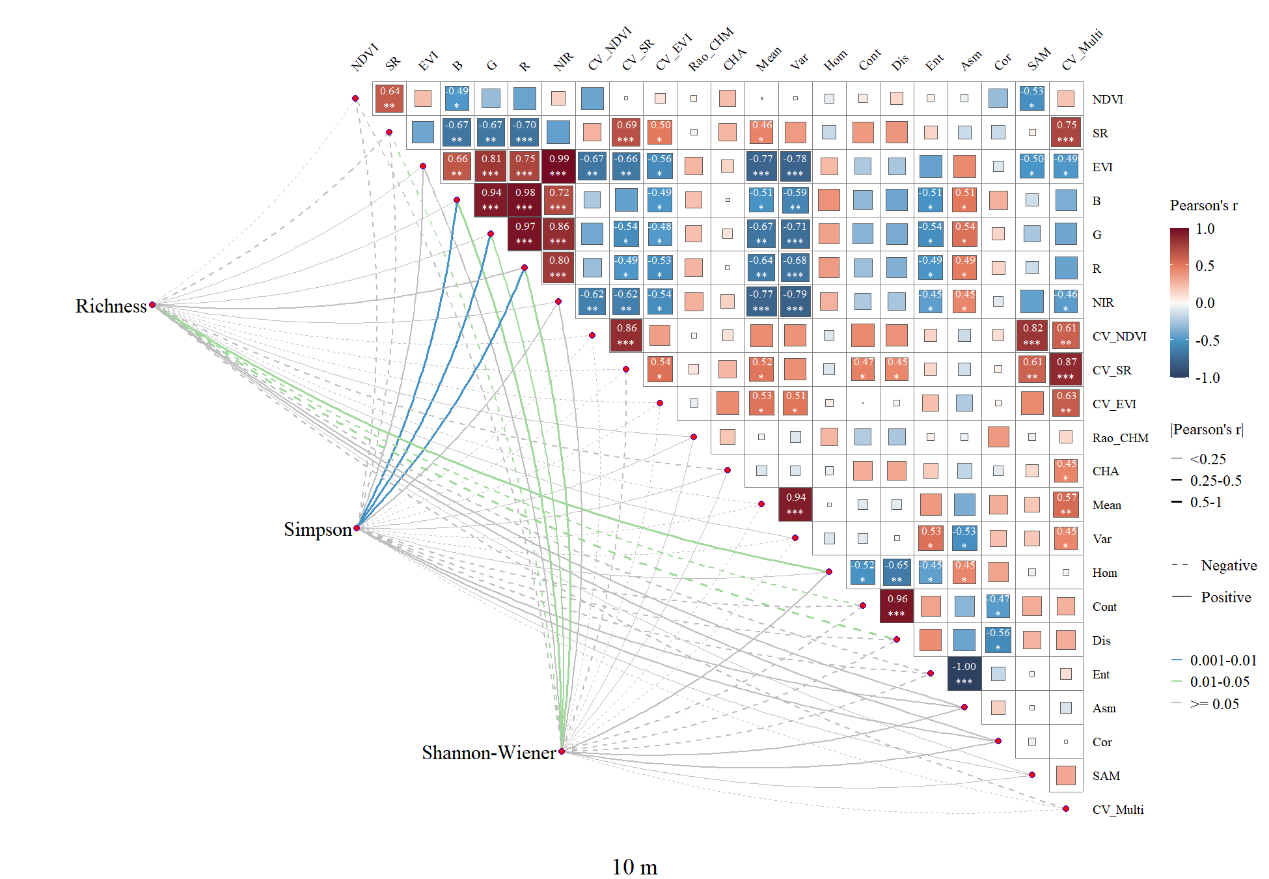


Supplementary Figure 13 Correlogram between in-situ species diversity and metrics based on 10 m image from GF2 and UAV LiDAR data.

## Supplementary Tables

Supplementary Table 1 In-situ species diversity metrics for 20 sample plots.

| Plot ID | Richness | Simpson | Shannon-Wiener |
| --- | --- | --- | --- |
| P01 | 16 | 0.491127 | 1.361564 |
| P02 | 17 | 0.8624 | 2.388658 |
| P03 | 9 | 0.486615 | 1.183816 |
| P04 | 13 | 0.875 | 2.312366 |
| P05 | 24 | 0.938272 | 2.998878 |
| P06 | 25 | 0.943985 | 3.084618 |
| P07 | 26 | 0.94625 | 3.101883 |
| P08 | 13 | 0.891967 | 2.40616 |
| P09 | 20 | 0.940972 | 2.925203 |
| P10 | 25 | 0.941043 | 3.03249 |
| P11 | 28 | 0.948846 | 3.160996 |
| P12 | 15 | 0.886719 | 2.452985 |
| P13 | 24 | 0.930839 | 2.938795 |
| P14 | 28 | 0.929752 | 3.05892 |
| P15 | 21 | 0.9375 | 2.913173 |
| P16 | 9 | 0.636837 | 1.515232 |
| P17 | 19 | 0.771267 | 2.13437 |
| P18 | 13 | 0.9056 | 2.456143 |
| P19 | 14 | 0.869684 | 2.329244 |
| P20 | 18 | 0.9 | 2.623683 |
